# RNA-Seq Signatures Normalized by mRNA Abundance Allow Absolute Deconvolution of Human Immune Cell Types

**DOI:** 10.1016/j.celrep.2019.01.041

**Published:** 2019-02-05

**Authors:** Gianni Monaco, Bernett Lee, Weili Xu, Seri Mustafah, You Yi Hwang, Christophe Carré, Nicolas Burdin, Lucian Visan, Michele Ceccarelli, Michael Poidinger, Alfred Zippelius, João Pedro de Magalhães, Anis Larbi

**Affiliations:** 1 Singapore Immunology Network (SIgN), Agency for Science Technology and Research, Biopolis, 8A Biomedical Grove, 138648, Singapore, Singapore; 2 Integrative Genomics of Ageing Group, Institute of Ageing and Chronic Disease, University of Liverpool, Liverpool L78TX, UK; 3 Department of Biomedicine, University Hospital and University of Basel, 4031 Basel, Switzerland; 4 Sanofi Pasteur, Marcy l’Etoile, France; 5 BIOGEM Research Center, Ariano Irpino, Italy; 6 Department of Science and Technology, University of Sannio, Benevento, Italy; 7 Department of Biology, Faculty of Sciences, University Tunis El Manar, Tunis, Tunisia; 8 Faculty of Medicine, University of Sherbrooke, Sherbrooke, QC, Canada; 9 Department of Microbiology, Immunology Programme, Yong Loo Lin School of Medicine, National University of Singapore, Singapore, Singapore

**Keywords:** immune system, flow cytometry, transcriptome, RNA-seq, gene modules, housekeeping, mRNA composition, mRNA abundance, mRNA heterogeneity, deconvolution

## Abstract

The molecular characterization of immune subsets is important for designing effective strategies to understand and treat diseases. We characterized 29 immune cell types within the peripheral blood mononuclear cell (PBMC) fraction of healthy donors using RNA-seq (RNA sequencing) and flow cytometry. Our dataset was used, first, to identify sets of genes that are specific, are co-expressed, and have housekeeping roles across the 29 cell types. Then, we examined differences in mRNA heterogeneity and mRNA abundance revealing cell type specificity. Last, we performed absolute deconvolution on a suitable set of immune cell types using transcriptomics signatures normalized by mRNA abundance. Absolute deconvolution is ready to use for PBMC transcriptomic data using our Shiny app (https://github.com/giannimonaco/ABIS). We benchmarked different deconvolution and normalization methods and validated the resources in independent cohorts. Our work has research, clinical, and diagnostic value by making it possible to effectively associate observations in bulk transcriptomics data to specific immune subsets.

## Highlights

• Characterization of 29 human immune cell type by RNA-seq and flow cytometry
• Modules of specific, co-expressed, and housekeeping genes are defined
• The mRNA heterogeneity and abundance are cell type specific
• The proposed normalization approach enables absolute deconvolution

## Graphical Abstract

**Figure:**
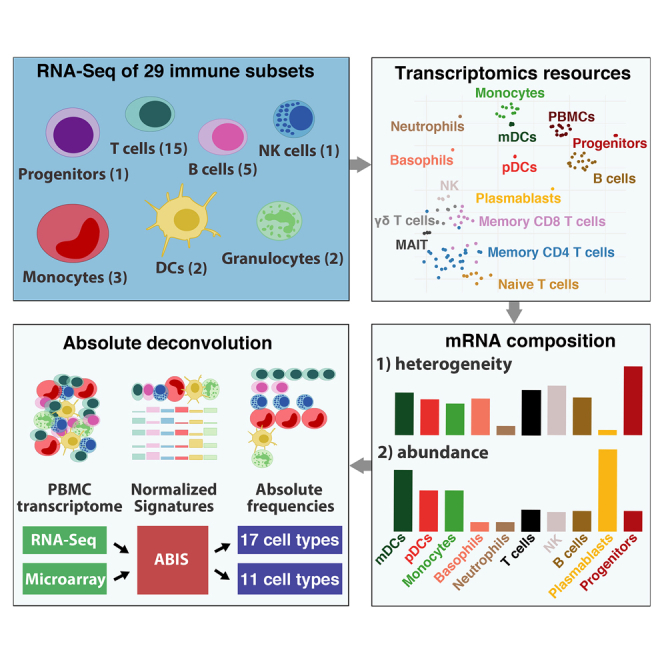

## Teaser

Monaco et al. generate an RNA-seq dataset on 29 immune cell types and identify modules of cell type-specific, co-expressed, and housekeeping genes. The mRNA heterogeneity and abundance of the different cell types were examined. Absolute deconvolution of PBMCs was obtained by taking into account mRNA abundance when normalizing the signature matrix.

## Introduction

The cellular heterogeneity of the immune system is essential for generating diverse and targeted immune responses. Because of ease of isolation and minimal invasiveness, investigations of the immune system are often limited to peripheral blood mononuclear cells (PBMCs). Vast amounts of transcriptomic data have been generated from the PBMC fraction (Corkum et al., 2015, van Leeuwen et al., 2005, de Mello et al., 2012, Miao et al., 2013); however, studying PBMCs in their entirety often contributes to results that are inconclusive or difficult to interpret, as it not always possible to accurately ascertain which specific immune cell types are responsible for any given transcriptomic signal of interest. Moreover, the proportion of immune cell subsets in the blood can vary during disease, age, or clinical interventions (vaccines and drugs), and these differences go undetected in the absence of data on immune cell composition.

A deconvolution approach can be an effective solution to discern specific immune cell type proportions from transcriptomic data of heterogeneous samples. Various deconvolution methods have been developed in the past decade (Shen-Orr and Gaujoux, 2013). Abbas et al. (2009) initially developed a deconvolution method that imposes two constraints on linear modeling (LM): sum to 1 and non-negativity (NNLM). A second approach is based on quadratic programming (QP) and was originally developed for microarray and later adapted for RNA sequencing (RNA-seq) data (Gong and Szustakowski, 2013, Gong et al., 2011). Newman et al. (2015) developed a method on the basis of support vector regression (SVR) that is more robust to noise and multicollinearity. More recently, several microarray datasets were collected to generate a signature matrix that is more robust to the gene expression platform used and individuals’ health conditions (Vallania et al., 2018),

Although the field of gene expression deconvolution has steadily grown since the first work reporting it (Lu et al., 2003), there are still several open questions that need to be addressed. First, deconvolution methods have been tested using mainly microarray data, which present limits in terms of signal resolution. RNA-seq data are increasingly becoming available for many immune cell types, but to our knowledge, there is no single comprehensive resource that encapsulates all the immune cell types of a heterogeneous immune sample together with ground-truth proportions needed for validation. Second, existing deconvolution methods rely on applying constraints in order to obtain absolute proportions instead of exploring different normalization strategies. Current normalization methods generally assume that cells have similar mRNA composition, and this can erroneously reduce or inflate deconvolution estimation for very different cells types. The cells composing the immune system show strong morphological and phenotypical differences, but their mRNA composition has not been examined in a systematic way yet. Third, previous works assumed that any cell type can be potentially deconvoluted. However, there are resolution limits imposed by the gene expression platform used and by the cell type mRNA landscape that have not been explored yet.

Here, we generated an RNA-seq gene expression profile of 29 immune cells constituting the PBMC fraction, together with fluorescence-activated cell sorting (FACS) proportions and gene expression of PBMCs. Transcriptomic analyses were performed to validate the dataset and to generate modules of genes that are specifically expressed in a cell type, co-expressed independently of cell lineage, and with housekeeping (HK) activity. Next, the mRNA composition in terms of abundance and heterogeneity was explored for our immune cell types. Last, we developed a normalization approach accounting for mRNA abundance that makes it possible to derive absolute proportions. We generated normalized signature matrices for a set of immune cell types that were found to be suitable for RNA-seq and microarray deconvolution of PBMC samples, respectively. Absolute deconvolution of external PBMC datasets can be directly applied using the Shiny app (https://github.com/giannimonaco/ABIS). The resources generated in this study will allow the dissection of molecular signatures at fine resolution and to quantitatively assess other state-of-the-art deconvolution methods.

## Results

### Detailed Characterization of 29 Immune Cell Types and PBMCs

**Figure 1:**
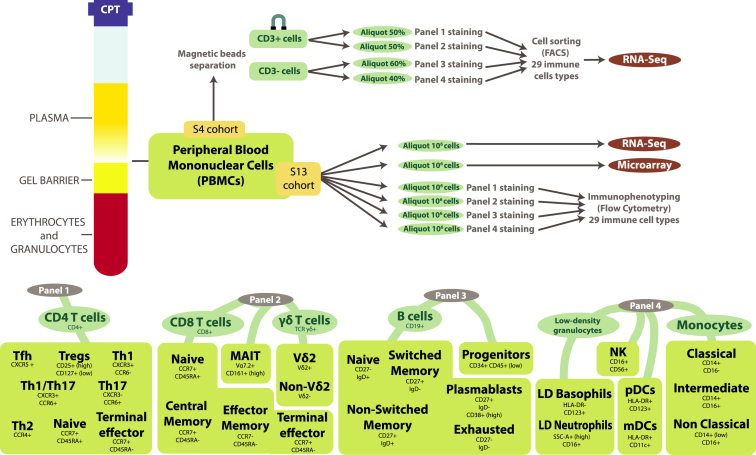
Representation of the Sample Preparation and Data Collection PBMC aliquots from two cohorts were used for (1) RNA-seq of 29 immune cell types (S4 cohort) and (2) microarray and RNA-seq of PBMCs and immunophenotyping of the 29 immune cell types (S13 cohort). Four staining panels (panels 1–4) were used to sort and immunophenotype the 29 immune cell types (Table S1). Tfh, T follicular helper; Tregs, T regulatory; Th, T helper; CE, central memory; EM, effector memory; TE, terminal effector; MAIT, mucosal-associated invariant T; SM, switched memory; NSM, non-switched memory; Ex, exhausted; LD, low-density; C, classical; I, intermediate; NC, non-classical; mDCs, myeloid dendritic cells; pDCs, plasmocytoid dendritic cells. See also Table S1 for full name and markers information.

Blood samples from four Singaporean individuals (S4 cohort) consisting of 29 immune cell types were sorted for transcriptomic profiling by RNA-seq. In addition, PBMC samples from a cohort of 13 Singaporean individuals (S13 cohort) were collected for PBMC transcriptomic profiling and flow cytometry-based immunophenotyping of the 29 immune cell types used for RNA-seq (Figures 1 and S1; STAR Methods; Table S1). The PBMC transcriptomic profiling of the S13 cohort was obtained by both RNA-seq and microarray technology in order to perform absolute deconvolution for both platforms.

The 29 immune cell types for this study were selected on the basis of their unique functionality and importance. The choice was also made with the aim of assigning each immune cell to a single cell type so that merging all the different cell types would reconstitute a complete PBMC sample. The 29 cell types included subsets of CD4 T cells (n = 8), CD8 T cells (n = 4) and B cells (n = 5), unconventional T cells (n = 3), natural killer (NK) cells (n = 1), monocytes (n = 3), dendritic cells (DCs) (n = 2), low-density (LD) granulocytes (n = 2), and progenitor cells (n = 1) (see STAR Methods for more details). The cell type with the lowest abundance was CD34+ hematopoietic progenitor cells (HPCs) (0.12%). See Table S1 for the mean and SD of the percentages of all cell types.

### Transcriptomics Analyses and Resources

In this section, we show various transcriptomics analyses to validate our dataset and to generate modules of immune-related genes which can be useful for future works.

#### Dimensionality Reduction and Clustering

**Figure 2:**
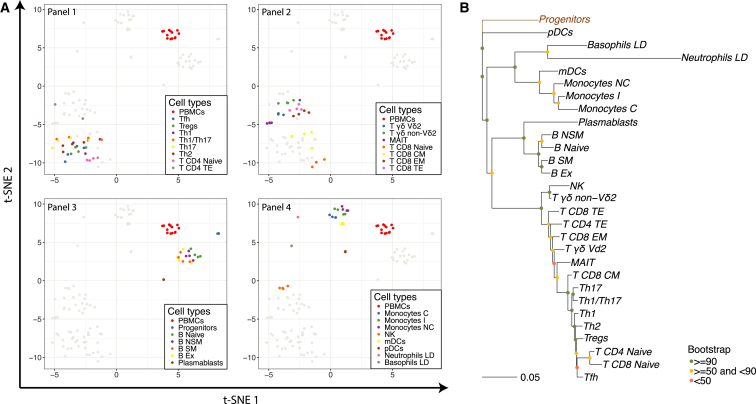
Relationship between Immune Cell Types, Determined Using log_2_ TPM Values (A) t-SNE analysis on the RNA-seq data of the 29 immune cell types and PBMCs. Results are shown in four separate plots to better distinguish the different cell types. Each plot highlights the PBMCs and the cell types of one of the four panels used for FACS. (B) Transcriptomic hematopoietic tree of the 29 immune cell types with progenitor cells fixed as the root of the tree.

We explored the ontogeny and relationships among the 29 immune cell types by applying dimensionality reduction and clustering algorithms to transcripts per million (TPM) expression values (Figures 2 and S2). The t-distributed stochastic neighbor embedding (t-SNE) analysis showed that for some cell types (progenitors, plasmablasts, LD neutrophils, LD basophils, and plasmacytoid DCs [pDCs]), samples obtained from different individuals grouped so closely that only one dot was visible in the plot (Figure 2A). The naive compartments of CD4 and CD8 T cells showed high similarity, and they clustered more closely together than with their corresponding memory subsets (Figures 2B and S2B). The T cell memory subsets formed two separate clusters: the CD4 T terminal effector (T_TE_) aggregated with the CD8 T effector memory (T_EM_) and CD8 T_TE_, and the CD8 T central memory (T_CM_) aggregated with the remaining CD4 T memory subsets (Figures 2A1, 2A2, S2A1, and S2A2). A closer look at the expression of genes related to degranulation activity, namely granzyme B (GZMB) and perforin (PRF1), revealed higher expression levels in CD4 T_TE_ compared with other identifiable CD4 T cell memory subsets, in accordance with previous results (Marshall and Swain, 2011).

The memory subtypes of T and B cells and intermediate (I) and non-classical (NC) monocytes showed poor specificity. Hierarchical clustering revealed that the gene expression signatures of these subtypes were more strongly influenced by inter-individual variability than by cell type differences (Figure S2B). A functional enrichment analysis revealed that the genes mainly responsible for individual variability were related to viral infection and type II interferon signaling.

#### Modules of Cell Type-Specific Genes

Cell type-specific genes were retrieved using both TPM and TPM_TMM_ values. The TPM values highlight the difference in gene expression proportions; the TPM_TMM_ gene expression values show the change in expression relative to a core set of genes. The differentially expressed genes (DEGs) were retrieved on the 29 cell types chosen for FACS and also on broader cell types (Table S2).

**Figure 3:**
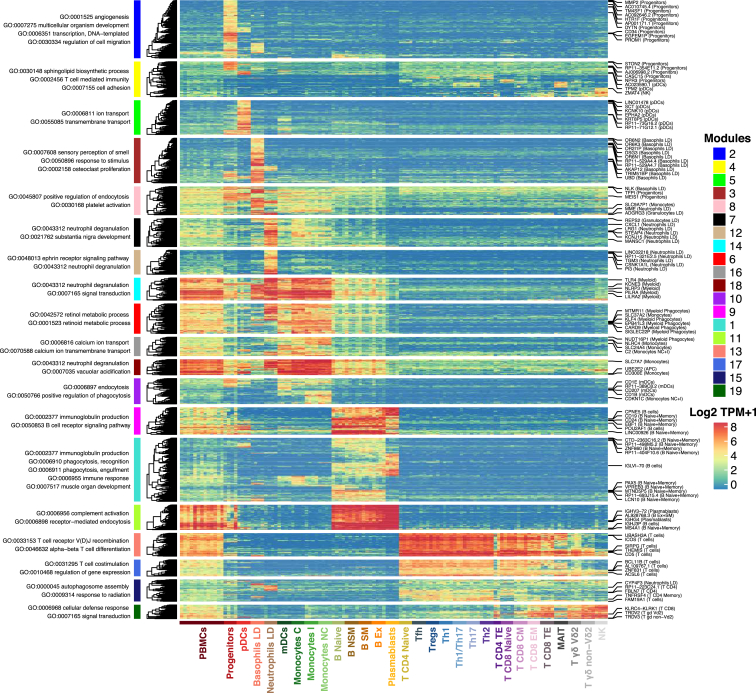
Heatmap of DEGs between Each Immune Cell Type and Remaining Samples Modules of genes were found by hierarchical clustering on Euclidean distance. The most biologically relevant GO terms associated with each module are reported on the left. The top differentially expressed genes (DEGs) are reported on the right. See the full list in Table S3.

Modules of cell type-specific genes were found by clustering the genes from the differential expression analysis (STAR Methods; Table S2). The heatmap of DEGs (Figures 3 and S3; Table S3) on the basis of TPM values confirms the quality of the transcriptomic data, as almost all cell types were enriched for their respective Gene Ontology (GO) terms. DEGs detected on the basis of TPM_TMM_ values (false discovery rate [FDR] < 0.05) were used to perform an enrichment analysis of gene sets from the Reactome database (Table S3). An example of significant pathways includes the enrichment of the mitotic cell cycle genes for plasmablasts and the downregulation of non-coding RNA activities for LD neutrophils (Table S3).

#### Modules of Co-expression Genes

We also retrieved modules of co-expressed genes (STAR Methods), which gather genes with similar expression patterns independently of cell type specificity (Figures S3 and S4). For each module we show the distribution of the connectivity values in Figure S3E. Some modules include genes broadly expressed in all or almost all cell types because they exert basic cell functions. For example, modules 8, 3, 11, and 7 are associated with transcriptional activity, and in addition, they have been found to be significantly enriched for transcription factors and co-factors listed in the AnimalTFDB (Zhang et al., 2015). Other modules include genes that exert a more specific immune function that can be carried out by multiple immune cell types. For example, module 13 is associated with antigen processing and presentation, which is done by B cells, monocytes, and DCs.

#### Immune Cell-Specific HK Genes

We explored the expression of HK genes retrieved from three publicly available lists (Eisenberg and Levanon, 2013, Hsiao et al., 2001, Tirosh et al., 2016). Although the overall SD of these HK gene lists was lower than the SD of the remaining genes, some discordant cases were identified. For example, the TPM_TMM_ values of commonly used HK genes *GAPDH* and *ACTB*, although expressed in all cells, were under-expressed in lymphoid cells and overexpressed in myeloid cells (Table S3). To find the appropriate threshold to identify immune-specific HK genes from our dataset, we checked the proportions of HK genes at different mean and SD thresholds using log_2_ TPM_TMM_ values (Figure S5). The expression of roughly 75% of HK genes had a mean > 5 and/or an SD < 1 (Table S3).

#### Comparison of Our Gene Expression Profiles with External Datasets

**Figure 4:**
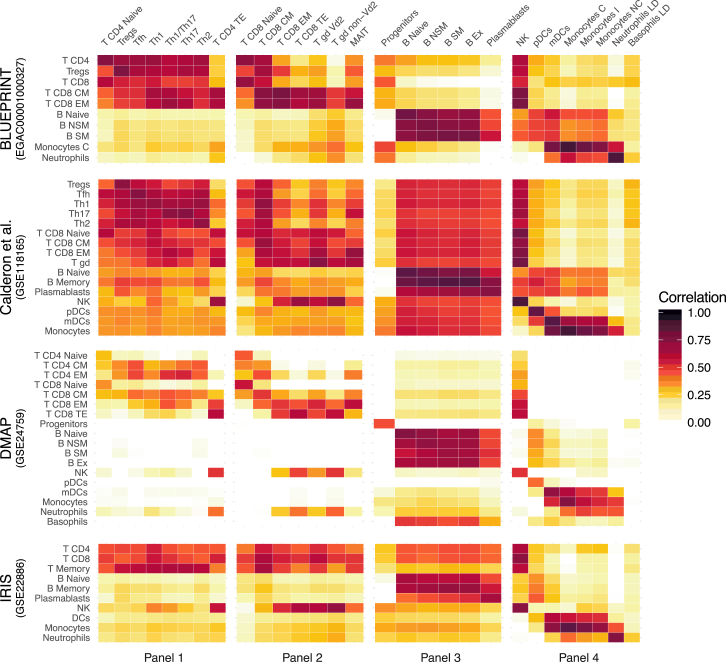
Comparison of the Gene Expression Profile of the Immune Cell Types from Our Dataset (Columns) with Four External Datasets (Rows) From the samples of each FACS panel in our dataset, we selected the top 1,000 variable genes and calculated the Spearman correlation with samples of external datasets. For the correlation, we used the cell type average of normalized expression values.

The gene expression profiles of our dataset were compared with external datasets in two ways (Figures 4 and S6A–S6D). The first way consisted of retrieving the top 1,000 most variable genes for the cell type of a FACS panel and performing Spearman correlation of the cell type average gene expression values of our dataset and an external dataset (Figure 4). Overall, the results indicate concordance between our dataset and the external dataset tested (Abbas et al., 2005, Novershtern et al., 2011).

The second way was to overlap the genes found to be specific for a cell type with our DEG analysis on TPM values with cell type markers described in previous works. A strong overlap exists with the DEGs found by Becht et al. (2016) and Abbas et al. (2005) (Figures S6A and S6B), but comparisons with Bindea et al. (2013) reveal a poor overlap among T cell subsets (Figure S6C). The single-cell RNA-seq study of Villani et al. (2017) was also used to compare DCs and monocytes; we found a good concordance as a stronger overlap was found between DC6 and pDCs and between myeloid DCs (mDCs) and DC2/DC3 (Figure S6D).

#### Using Our Cell Type-Specific and Co-expression Modules to Analyze an Influenza Vaccine Cohort

The differential expression and co-expression data were used to analyze a microarray dataset of PBMCs that was collected for studying the immune response to influenza vaccination at four different time points (0, 2, 7, and 28 days). We performed pairwise comparisons of the data at days 2, 7, and 28 versus day 0 (the baseline time point), and we identified the co-expression modules previously retrieved (Table S3) that were enriched at each time point (Fisher’s test with p value < 0.05) (Figure S6E; Table S4).

Genes that were upregulated on day 2 were associated with activation of the innate immune response, and downregulated genes were associated with T cell activation. For day 7 post-vaccination samples, there was an enrichment for genes specific for antibody secreting cells, particularly plasmablasts, and concordantly, enriched modules were related to B cell signaling, cell cycle, and protein folding. As expected of typical immune kinetics during vaccination, we found no significant upregulation of co-expression modules on samples from day 28, indicating a reversion to baseline profiles. Figure S6E also shows which are the transcription factors and co-factors as they might be responsible for the observed transcriptional changes.

### Transcriptome Composition

Here, we explored the transcriptome composition of our 29 immune cell types which belong to different lineages with large phenotypical and morphological differences. These findings highlight limitations in using normalization methods which assume similar mRNA composition in different cell types.

#### mRNA Heterogeneity

With the TPM normalization, expression values are scaled so that their sum is always 10^6^ for each sample. This approach allows transcript proportions to be comparable among samples. However, in case the total mRNA of a sample is dominated by the expression of only a few genes, the remaining fraction of genes will be characterized by especially low expression values. This effect applies only to RNA-seq data, not to microarray data, as RNA-seq does not have an upper limit in its dynamic range (Bullard et al., 2010a).

**Figure 5:**
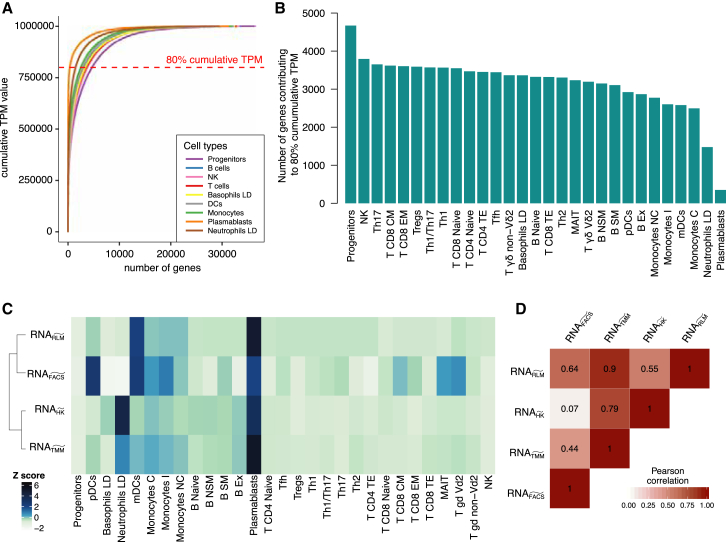
Two Aspects of mRNA Composition: Heterogeneity and Abundance (A and B) Heterogeneity. (A) The cumulative sum of the median TPM values of nine relevant cell types calculated from values sorted in decreasing order. The total sum of TPM values is always 10^6^. (B) The minimum number of genes that contribute to 80% of total gene expression in the 29 cell types. This number corresponds to the dashed red line in (A). (C and D) Abundance. (C) mRNA scaling factors for the 29 immune cell types calculated with four methods (STAR Methods). For the clustering distance between rows, we used the Spearman correlation. (D) Pearson correlation matrix for the values reported in (C).

The comparison of cumulative TPM expression between different immune cell types makes it possible to identify profound differences in the mRNA composition with regard to transcript heterogeneity. For example, in plasmablasts and LD neutrophils, we found that relatively few genes were responsible for the largest fraction of total mRNA (Figures 5A and 5B). A contrasting observation was made for progenitor cells, which had the greatest diversity of expressed genes, an outcome that likely stems from their lack of commitment to specialized functions (Kingsley et al., 2013). These findings also explain why data from plasmablasts and LD neutrophils exist at a substantially different scale from the other immune subsets (Figures 3, S4, and S7A).

#### mRNA Abundance

The observation that both plasmablasts and LD neutrophils display low mRNA heterogeneity does not imply similar mRNA composition. Therefore, a second factor that must be considered is total mRNA abundance, which can vary greatly among cell types because of two main factors that correspond either to cell size or metabolic activity. For example, active cell cycling requires increased metabolic activity, which correlates with increased mRNA abundance (Tanenbaum et al., 2015).

By dividing the total RNA yield obtained from the RNA quantification assay (STAR Methods) by the corresponding number of cells obtained from cell sorting, we could estimate the RNA yield per cell for each cell type (RNA_FACS_). Our results indicate high RNA yield for plasmablasts, DCs, and monocytes and low RNA yield for LD granulocytes, progenitor cells, and CD4 T_TE_ (Figures 5C and S7B).

We then calculated scaling factors that should mainly correct for mRNA abundance when applied to TPM values (as they are already normalized by RNA-seq library). The scaling factors are values that optimize the error between deconvolution results and flow cytometry proportions (Figure 7C; STAR Methods and Absolute Deconvolution), inverted HK mean values (Figure S7D), and inverted TMM values (RNA_TMM_) (Robinson and Oshlack, 2010) (Figure S7E; STAR Methods). When comparing the values generated by the different approaches (Figures 5C and 5D), we noticed an ostensible discordance for a few cell types, particularly LD neutrophils. As discussed, LD neutrophils have a few highly expressed genes that crowd the total mRNA pool. Hence, TMM and HK methods may overestimate the mRNA scaling factor in attempting to normalize the expression of core gene sets (the majority of genes) across all cell types. However, the total RNA output of LD neutrophils is lower than that of many other immune cells, as indicated by our RNA_FACS_ estimation. This finding suggests that certain normalization methods, such as the alignment of HK gene expression, upper quartile (UQ) (Bullard et al., 2010a), TMM (Robinson and Oshlack, 2010), and relative log expression (RLE) (Anders and Huber, 2010) should be avoided if the aim is to normalize for mRNA abundance across very diverse cell types.

### Absolute Deconvolution

Here, we used RNA-seq data to perform absolute deconvolution on a suitable set of immune cell types using a procedure that derives scaling factors for mRNA abundance normalization. This same approach was thereafter adapted to be used with microarray data.

#### Search for the Most Suitable Cell Type Combination

**Figure 6:**
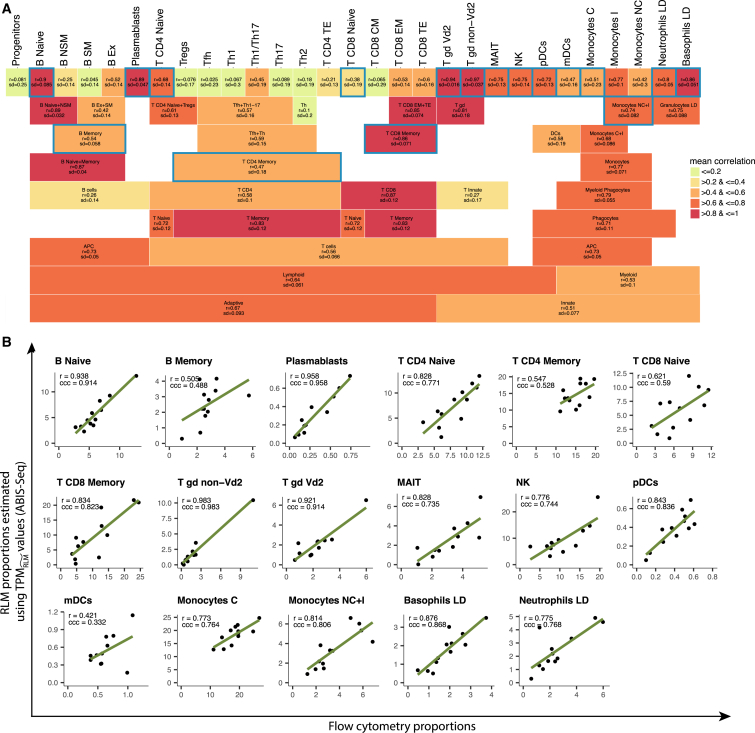
Absolute Deconvolution of RNA-Seq PBMC Samples (A) Exhaustive search for cell types that are suitable for deconvolution from PBMC-derived RNA-seq data. For each cell type, we report the mean and SD of Pearson correlations obtained by deconvolution of all possible combinations of cell types (merged and non-merged) that reconstitute a PBMC sample. Cell types that have been chosen for the deconvolution analysis in (B) are outlined in blue. (B) Comparison of deconvoluted and flow cytometry proportions on 17 immune cell types with respect to PBMCs. The concordance correlation coefficient (ccc) and the Pearson correlation coefficient (r) are shown on each plot.

Deconvolution methods work only for cell types that have detectable and distinctive signals from a heterogeneous sample. Hence, we performed a preliminary exhaustive search on all cell types created by merging all possible combinations of T cell, B cell, and monocyte subtypes. This was done by generating the Pearson correlation between the deconvoluted and flow cytometry proportions for all the merged cell types. From the preliminary exhaustive search, we delineated nine classifications (the ones also used for DEG analysis), which include the cell types that yielded the highest mean Pearson correlation. Next, we performed a second exhaustive search using the cell types of the nine classifications to obtain a unique well-performing cell type classification for deconvolution (Table S5), that is, 17 cell types for RNA-seq (Figure 6) and 11 cell types for microarray (Figure S8).

#### mRNA Normalization through RLM Deconvolution and Optimization

Deconvolution requires absolute expression values, which is in contrast to differential expression analysis, for which it might suffice to compare counts normalized only for library size. For example, in the case of LD neutrophils, it is undesirable to increase the total gene expression values if the mRNA abundance is relatively low compared with the remaining cell types. Hence, one way to normalize RNA-seq data for deconvolution approaches is to calculate TPM values first, followed by multiplying these values with a scaled mRNA abundance value.

Although obtaining TPM values is straightforward, normalizing for mRNA abundance can be tedious. We demonstrated the impossibility of relying on certain mathematical methods (e.g., 1/TMM) to obtain absolute measurements (Figures 5C and 5D). Moreover, it is preferable not to use the total RNA yield estimates from our RNA quantification divided by FACS enumeration for two reasons: (1) the quantification has been made on total RNA, and (2) the estimate is accurate only for a limited dynamic range (1–200 ng).

Hence, we outlined a method to estimate scaling factors that normalize TPM values for mRNA abundance using a robust deconvolution method that works without constraints, that is, robust LM (RLM) and a one-dimensional optimization procedure. In our method, we first built a signature matrix including a set of predictor variables (cell types) so that their merging reconstitutes a full PBMC sample. Second, we used RLM to estimate β coefficients from PBMC-derived transcriptomics data (the response variable) and immune cell types (the predictor variables). As we use TPM values, the β coefficients that were derived by RLM embody both the contributions of immune cell proportions and mRNA abundance. Hence, as a final step, we isolated the latter component by using an optimization procedure that locates a value that minimizes the error between estimated and real cell type proportions (STAR Methods). This RLM deconvolution and optimization procedure was performed using a well-conditioned signature matrix derived from the 4 individuals of the S4 cohort and on the flow cytometry and RNA-seq PBMC data of 12 individuals from the S13 cohort. The patterns of the estimated mRNA scaling factors were relatively closer to those obtained by RNA quantification and FACS enumeration as shown before (Figure 5C).

#### Absolute Deconvolution for RNA-Seq PBMC Samples

When validating deconvolution, a high Pearson correlation coefficient (r) indicates only that specific signal is present in the signature matrix to allow the accurate estimation of alterations in cellular proportions. However, to reveal if a robust estimation of absolute numbers was obtained, a high concordance correlation coefficient (ccc) must be attained. Figure 6B shows the results obtained for the deconvolution of 17 immune cell types using a signature matrix that has been normalized for mRNA abundance with scaling factors that were derived as described above (ABIS-seq [absolute immune signature for RNA-seq]). Because we used a method that is robust to noise (i.e., RLM), we did not filter out any noisy gene. For all cell types, we observed a less than 0.1 difference between the Pearson and concordance coefficients (Figure 6B). Our signature matrix, ABIS-seq, can be directly used on TPM values of external RNA-seq data of PBMC samples (see ABIS-seq in Table S5).

#### Absolute Deconvolution for Microarray PBMC Samples

Deconvolution was then performed using microarray data for the same PBMC samples used for the RNA-seq deconvolution (S13 cohort). For the cross-platform normalization, we kept only the genes that yielded a Pearson correlation of >0.70 (755 genes) between the matching RNA-seq and microarray data. From the selected genes, a scaling factor was calculated by dividing the UQ of microarray genes with the UQ of RNA-seq genes. The microarray data from each sample was then divided by the corresponding scaling factor.

The signature matrix used for microarray deconvolution was filtered of noisy genes, that is, very high or low as well as non-specific genes (STAR Methods). In contrast to RNA-seq, we noticed that even a robust method such as RLM produced poorer deconvolution results. However, we still obtained reasonable cccs between estimated and ground-truth proportions for several cell types (>0.8 for naive B cells and mDCs; >0.6 for T naive, monocytes, LD neutrophils, and LD basophils) (Figure S8B). The signature matrix (ABIS-microarray) and the target quintiles to normalize the ABIS-microarray gene set of external PBMC samples are available in Table S5.

#### Benchmark of Five Deconvolution Methods

**Figure 7:**
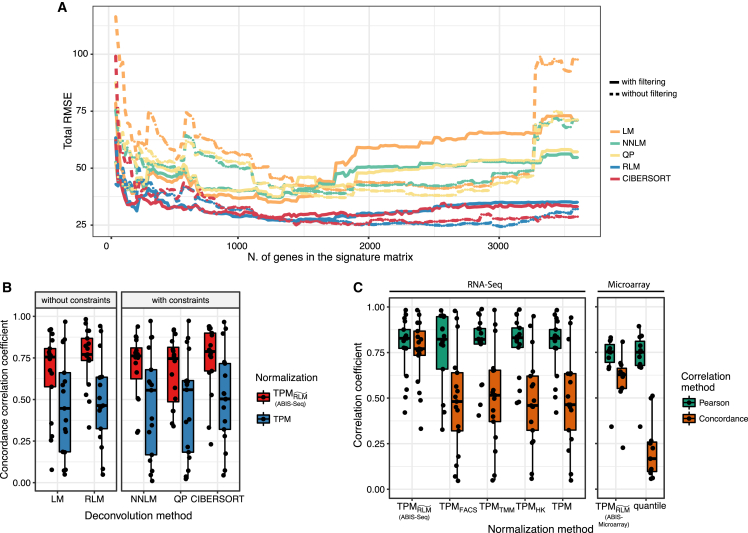
Benchmarks and Validations of Different Deconvolution and Normalization Methods (A) Comparison of five deconvolution algorithms in the presence and absence of noise and at increasing size of the signature matrix. The total RMSE is calculated by using the estimated and ground-truth proportions of the 17 cell types of RNA-seq deconvolution. (B) Comparison of results obtained from deconvolution methods with and without constraints and using our signature matrix for RNA-seq deconvolution with either TPM values or absolute expression values (ABIS-seq). (C) Comparison of RNA-seq and microarray deconvolution results with different normalization methods. Each dot is a different cell type.

We compared the performance of five different deconvolution methods (Figure 7A) using our RNA-seq dataset. The five deconvolution methods compared are LM, non-negative LM (NNLM) (Abbas et al., 2009), RLM, QP (Gong et al., 2011), and SVR as used for CIBERSORT (Newman et al., 2015). The performance of each method was evaluated using the root-mean-square error (RMSE) obtained between deconvoluted and flow cytometry proportions. Noise and multicollinearity were respectively evaluated by the absence of gene filtering and by increasing the number of genes for the signature matrix. The gene-filtering procedure again consisted of removing genes with very low and high expression as well as those that lack specificity (STAR Methods). Among the five methods, our study shows that CIBERSORT and RLM are least affected by both noise and multicollinearity. However, all deconvolution methods also performed relatively well with a filtered and a well-conditioned signature matrix.

The ability of deconvolution methods that implement constraints to give better estimates was then evaluated (Figure 7B). We compared the deconvolution results obtained from TPM and ${\text{TPM}}_{\tilde{\text{RLM}}}$ with methods that apply no constraints (LM and RLM) and with three methods that apply constraints (NNLM, QP, and CIBERSORT). As hypothesized, we found that applying constraints is not sufficient to obtain absolute estimates. In fact, the cccs were substantially lower when using TPM expression values compared with using ${\text{TPM}}_{\tilde{\text{RLM}}}$ independently of the deconvolution method used.

#### Validation of Our Normalization Method and Signature Matrices

The RNA-seq and microarray deconvolution analyses were repeated using different normalization strategies, which are TPM, TPM_FACS_, TPM_HK_, and TPM_TMM_ for RNA-seq and quantile normalization for microarray. The Pearson correlation values between estimated and real proportions remained high across all normalization methods. However, the cccs remained high only for ${\text{TPM}}_{\tilde{\text{RLM}}}$, while it drastically dropped for all the other normalization strategies (Figure 7C).

Last, we tested absolute deconvolution using our normalized signature matrices (Table S5) on external RNA-seq and microarray datasets (Mohanty et al., 2015, Newman et al., 2015, Zimmermann et al., 2016) (Figure S9A). Moreover, for comparison we performed deconvolution on quantile normalized signature matrices including results obtained using three independently proposed signature matrices: IRIS (Abbas et al., 2005, Abbas et al., 2009), LM22 (Newman et al., 2015), and ImmunoStates (Vallania et al., 2017) (Figure S9B). Our signature matrices normalized for mRNA abundance performed consistently better in all cases (Figure S9).

## Discussion

In this study, we generated and analyzed gene expression and flow cytometry data for 29 immune cell types constituting the PBMC fraction. The data were mainly used to address open questions on the deconvolution of heterogeneous immune samples, although it was also used for the generation of valuable resources. The patterns of the transcriptomics dataset were revealed by using dimensionality reduction and clustering methods (Figures 2 and S2). Highly distinct profiles were obtained for LD neutrophils, LD basophils, plasmablasts, progenitors, and pDCs; the remaining cell types grouped within broader categories with varying degrees of overlap. For example, CD8 T cells with effector functions, such as CD8 T_EM_ and CD8 T_TE_, clustered closely together, as expected from previous findings (Willinger et al., 2005). These subtypes were also closely related to CD4 T_TE_ cells and other cell types with degranulation activity, such as non-classical T cells and NK cells. A separate group of T cells consisted of CD4 memory T cells and CD8 T_CM_ cells. As previously reported (Pennock et al., 2013), these cells can be distinguished by their strong cytokine production capacity. Finally, T cells with a naive phenotype formed an independent cluster, regardless of their commitment to the CD4 or CD8 lineage.

The gene expression data were then used to retrieve sets of differentially expressed, co-expressed, and immune-specific HK genes (Table S3). Enrichment analysis using the GO and Reactome databases revealed the functionalities of modules of genes. As our data are at RNA-seq resolution, it may be possible to identify (by association) candidate genes that have unknown or partially known functions. Our modules can also be used to enrich gene expression analyses from datasets produced at the microarray level, as demonstrated by the analysis of our vaccination cohort (vaccine cohort) (Figure S6E).

Among the 29 immune cell types, the transcriptome composition was particularly different in progenitors, LD neutrophils, and plasmablasts. Progenitors revealed the largest heterogeneity of gene expression as a wide spectrum of mRNA molecules are produced by its transcriptional machinery. In contrast, LD neutrophils and plasmablasts have very few specific genes that contribute greatly to the total mRNA composition (Figures 5A and 5B). Although both plasmablasts and LD neutrophils have a relatively low mRNA heterogeneity, their mRNA abundance is at two different extremes in comparison with the other immune cell types (Figures 5C and 5D).

The normalization for mRNA abundance in differential expression analyses can lead to misleading results. For example, if the analysis is done with absolute values of two cell types with largely different total mRNA output (e.g., 100 for cell type A and 1,000 for cell type B), this would probably lead to the erroneous conclusion that all the genes in cell type A are downregulated. However, normalizing for mRNA abundance is critical for the purposes of deconvolution. Existing methods that include the UQ, TMM, and RLE (Anders and Huber, 2010, Bullard et al., 2010a, Robinson and Oshlack, 2010) cannot correctly identify cases in which the overall transcriptional machinery is downregulated or upregulated. Similarly, this happens when attempting to normalize gene expression by using HK genes as reference whose expression levels are assumed to be similar among different cell types (Risso et al., 2014). This led us to develop an approach that consisted of scaling the TPM values by a factor that minimizes the error between flow cytometry and deconvoluted proportions $\left({\text{TPM}}_{\tilde{\text{RLM}}}\right)$. We used RLM to deconvolute the cell type proportions, which is a method with no constraints and robust to noise (STAR Methods). The procedure needs to be performed only once to normalize the signature matrix for a type of heterogeneous sample. Hence, the provided two signature matrices, one for RNA-seq and one for microarray data (ABIS-seq and ABIS-microarray), can be directly used for PBMC deconvolution (Table S5).

Deconvolution is accurate only if it can detect a signal that is specific for a cell type and that is expressed in a consistent pattern among the cell types of a heterogeneous sample. Therefore, when needed, we merged the sorted cell types into broader cell types with a specific signal that is detectable from PBMC expression data. For RNA-seq, we obtained optimal results by using 17 cell types. Specifically, we combined the memory subsets of B cells and T cells as well as the non-classical and intermediate monocytes (Figure 6A). Progenitor cells were the only cell type not suitable for deconvolution that could not be grouped with other cell types. The deconvolution results after normalization for mRNA abundance were robust even for cell types that existed at very low frequencies within PBMCs, such as pDCs, mDCs, LD neutrophils, and LD basophils.

For microarray deconvolution we obtained optimal results for 11 cell types, that were then used to retrieve mRNA scaling factors with our deconvolution and optimization approach (Figure S8). Overall, the results were less accurate in comparison with the deconvolution results obtained with RNA-seq data. We attribute this difference to two main disadvantages of the microarray platform: (1) an imposed upper limit due to probe saturation (Gong et al., 2011) and (2) the measurement of gene expression levels on a limited set of pre-annotated genes. For example, data on *TRDV2* gene expression, which is essential for deconvoluting the signal from Vδ2 T cells, were absent. A shared limitation between both microarray and RNA-seq technologies is the susceptibility of low gene expression signals to background noise, which seemed to be the most plausible explanation for the poor deconvolution of progenitor cells. This limitation, however, can be potentially circumvented for RNA-seq data by increasing sequencing depth. In this perspective, PBMCs might be more informative than whole blood, in which neutrophils constitute approximately 40%–80%, and it would more likely obfuscate the signal of other cell types. Nevertheless, the deconvolution of whole blood should be investigated in future studies as it represents an untouched source of biological samples.

Although RLM was used for all the deconvolution analyses, several other deconvolution algorithms have been made available in recent years (Abbas et al., 2009, Gong et al., 2011, Newman et al., 2015, Shen-Orr and Gaujoux, 2013). We assessed the performance of five of these deconvolution methods (Figure 7A) and found that RLM and SVR, as used in CIBERSORT (Newman et al., 2015), were least affected by noise and multicollinearity. Moreover, all tested methods achieved optimal performance when a filtered and well-conditioned signature matrix was used. Nevertheless, we rationalized that it was more useful to adopt a method that was unconstrained (such as LM or RLM) in exploratory phases because they have a tendency to reveal sources that generate noise within a dataset. Moreover, we demonstrated that using constraints, such as non-negativity and total sum to 1, does not improve absolute estimation if data are not properly normalized for mRNA abundance (Figure 7B).

Our normalization approach outperforms commonly used normalization approaches in the estimation of absolute proportions (Figure 7C). This was also tested in external datasets and compared with the results obtained using signature matrices produced in previous works (Figure S9). The external validation could be performed only on major cell types, because of the lack of ground-truth data for finer cell types. Moreover, this also allowed a fairer comparison with external signature matrices, as they are all designed to deconvolute a different set of immune cell types. However, a more comprehensive benchmark should be performed when more data become available. This should be especially done for low-frequency cells which we found not suitable for deconvolution but were included in other signature matrices (such as the Tfh, Tregs, and T gd in LM22).

Several issues deriving from technical and biological variability should be considered when generating a signature matrix. Technical factors that may interfere with the deconvolution analysis include sample preparation protocol, cell isolation method, and transcriptomics platform used. For example, flow cytometry has several limitations, as it suffers from spectral overlap, it produces background signal, and it induces cellular stress or even cellular death on especially susceptible cells, such as neutrophils (Hu et al., 2016). This affects both the calculation of the ground-truth cell proportions and the gene expression profile of sorted cells. Other approaches, however, are not free from limitations. All methods that use antibodies as a way to detect a target molecule, such as immunohistochemistry (IHC), magnetic-activated cell sorting (MACS), and mass cytometry, are biased by the binding efficiency of the clones used (Ivell et al., 2014). In addition, the labeling of an antibody with fluorophores, metals, or beads can modify the binding specificity of the antibody (Atkuri et al., 2015). More specifically, mass cytometry cannot be used to sort cells, as it disintegrates the cells analyzed, and there are additional contaminating sources that must be considered, such as metal impurities and oxidation products (Leipold et al., 2015). MACS induces less stress than FACS in sorted cells, but it generally gives lower purity and it does not provide cell percentages (Hu et al., 2016, Li et al., 2013).

Biological factors that may contribute to cohort-specific observations include gender, age, ethnicity, and pathological condition. For example, a pathologic condition could drastically alter the total mRNA abundance of certain immune subsets or the expression of genes believed to be specific to one cell type. In extreme cases, different biological settings could introduce new subtypes that would generate noise, as these were absent in our original PBMCs samples. Hence, generating a single signature matrix that is robust to individuals’ health conditions and gene expression platform, as done in a recent work (Vallania et al., 2018), might reduce the performance that could be obtained in specific settings. However, this kind of meta-analysis in which many different datasets are collected is necessary to better understand the limitations of deconvolution.

In conclusion, using RNA-seq data from 29 different immune cell types, we comprehensively explored the transcriptomics pattern and signature of each immune cell type, thereby generating a library of transcriptomic resources, including DEGs, co-expressed genes, and immune-specific HK genes. In addition, we took into consideration and revealed detailed differences among the various immune subsets for two aspects of mRNA composition: mRNA heterogeneity and mRNA abundance. Last, we developed a method for normalizing RNA-seq data for mRNA abundance to enable absolute deconvolution. The same method was also adapted for microarray deconvolution. We provide the signature matrices and a Shiny app to directly perform deconvolution of PBMC gene expression data (https://github.com/giannimonaco/ABIS). This work raises new questions and possibilities as to how immune gene expression data can be analyzed to generate information, not only for future studies but also for completed ones. We believe that our work provides greater dimensionality to the current landscape of immunogenetic research and makes a relevant step into understanding and devising strategies to tackle immunological phenomena.

## STAR★Methods

### Key Resources Table

**Table:** 

REAGENT or RESOURCE	SOURCE	IDENTIFIER
**Antibodies**		

CD3 Microbeads human	Miltenyi Biotec	Cat#130-050-101
Panel 1 to 4	Table S1 – This paper	N/A
Panel 5	Table S1 – This paper	N/A

**Biological Samples**		

Human blood samples	SIGN, A^∗^STAR	NUS-IRB 10-250

**Critical Commercial Assays**		

CPT™	BD Biosciences	Cat#362761
TRIzol® reagent	Thermo Fisher Scientific	Cat#15596026
mirVana isolation kit	Thermo Fisher Scientific	Cat#AM1560
Quant-iT™ RiboGreen® RNA Assay Kit	Thermo Fisher Scientific	Cat#R11490
TargetAmp 2-Round aRNA Amplification Kit 2.0	Epicenter	Cat#TAU2R51224
Illumina® TotalPrep RNA Amplification Kit	Thermo Fisher Scientific	Cat#AMIL1791

**Deposited Data**		

GENCODE v26	Harrow et al., 2012	https://www.gencodegenes.org, RRID:SCR_014966
Reactome v61	Fabregat et al., 2016	https://reactome.org, RRID:SCR_003485
RNA-Seq of 29 immune cell types and PBMCs of the S4 and S13 cohort, respectively	This paper	GEO: GSE107011
Microarray of PBMCs of the S13 cohort	This paper	GEO: GSE106898
Microarray of PBMCs of the vaccine cohort	This paper	GEO: GSE107990

**Software and Algorithms**		

FACSDiva v6	BD Biosciences	RRID:SCR_001456
FlowJo v10	FlowJo, LLC	https://www.flowjo.com/solutions/flowjo; RRID: SCR_008520
flowAI v1.4.2	Monaco et al., 2016	10.18129/B9.bioc.flowAI
FastQC v0.11.5	Babraham Institute	http://www.bioinformatics.babraham.ac.uk/projects/fastqc/, RRID:SCR_014583
Kallisto v0.43.1	Bray et al., 2016	https://pachterlab.github.io/kallisto/download
Tximport v1.6.0	Soneson et al., 2016	10.18129/B9.bioc.tximport
MultiQC v1.0	Ewels et al., 2016	http://multiqc.info RRID:SCR_014982
ComBat (from sva v3.26.0)	Johnson et al., 2007	10.18129/B9.bioc.sva; RRID:SCR_012836
ggplot2 v2.2.1	Wickham, 2009	http://ggplot2.org/; RRID:SCR_014601
limma v3.34.9	Ritchie et al., 2015	10.18129/B9.bioc.limma; RRID:SCR_010943
EDAseq v2.12.0	Risso et al., 2011	10.18129/B9.bioc.EDASeq; RRID:SCR_006751
Rtsne v0.13	Jesse Krijthe	https://cran.r-project.org/web/packages/Rtsne/index.html
ape v5.0	Paradis et al., 2004	https://cran.r-project.org/web/packages/ape/index.html
WGCNA v1.63	Langfelder and Horvath, 2008	https://cran.r-project.org/web/packages/WGCNA/index.html RRID:SCR_003302
dynamicTreeCut v1.63-1	Langfelder et al., 2008	https://cran.r-project.org/web/packages/dynamicTreeCut/index.html
ComplexHeatmap v1.17.1	Gu et al., 2016	10.18129/B9.bioc.ComplexHeatmap
Non-linear least square regression (NLLSR)	Abbas et al., 2009	N/A
QP	Gong et al., 2011	N/A
CIBERSORT	Newman et al., 2015	https://cibersort.stanford.edu/
ABIS deconvolution	This paper	https://github.com/giannimonaco/ABIS

### Contact for Reagent and Resource Sharing

Requests for further information should be directed to and will be fulfilled by the Lead Contact, Dr. Gianni Monaco (mongianni1@gmail.com).

### Experimental Model and Subject Details

#### Donors

Blood from four Singaporean healthy individuals (2 males and 2 females) aged 20-35 years (S4 cohort) was collected for RNA-Seq transcriptomic profiling of the selected 29 immune cell types. Blood from the S4 cohort and from a further nine Singaporean healthy individuals (7 males and 2 females) aged 20-35 years (S13 cohort) was used for flow cytometry-based immunophenotyping of the 29 immune cell types, and microarray and RNA-Seq transcriptomic profiling of PBMCs (Figure 1). The study was approved by the NUS Institutional Review Board (IRB number NUS-IRB 10-250). All subjects gave informed consent and samples were pseudo-anonymized. To reduce variability, blood was drawn in the morning from fasting participants and there were no changes in the personnel involved in performing the experiments. Analyses on gender differences were not performed in this work as a larger cohort is needed in order to obtain statistically robust results. For validation analyses, we used a vaccine cohort from an in-house clinical trial NCT: NCT03266237. In this study, blood was collected from 240 Singaporean individuals aged 23-89 years on day 0, 2, 7, and 28 following flu vaccination. These samples were used for flow cytometry immunophenotyping and microarray transcriptomic profiling.

### Method Details

#### Antibody panel design

Four antibody staining panels were designed to sort (cohort S4) and immunophenotype (cohort S13) the 29 immune cell types from the following broad categories: 1) CD4 T cells (panel 1); 2) CD8 T cells, mucosal associated invariant T (MAIT) cells and γδ T cells (panel 2); 3) B cells and progenitor cells (panel 3); and 4) monocytes, NK cells, DCs and LD granulocytes (panel 4). The 29 cell types were chosen to cover the majority of cells that constitute a PBMC sample without any overlap among cell types (Figures 1 and S1 and Table S1).

For panel 1, we used chemokine receptors to distinguish between T helper subtypes (Brodie et al., 2013, Brodie et al., 2016). From the results given by the two papers of Brodie et al. and Milteny guidelines we decided to use the chemokine markers, CCR6, CXCR3 and CCR4, to discriminate between Th1, Th2, Th17 and Th1/17. We did not consider Th9 and Th22 as they are generally present in very low percentages in the blood and it was impractical for us to include more cell types in panel 1. Because most Th cells are central and effector memory CD4 T cells (CM and EM), we used CCR7 and CD45RA to isolate naive and terminal effector (TE) CD4 T cells in order to fill the CD4 T cell compartment. The T follicular helper (Tfh) cells were recognized by their specific expression of chemokine marker CXCR5 (Mahnke et al., 2013b, Crotty, 2011). The discrimination of T regulatory cells follows the suggestions proposed in previous works (Liu et al., 2006, Mahnke et al., 2013a).

For panel 2, we used CCR7 and CD45RA to classify the CD8 T cells across four maturation stages: naive, central memory (CM), effector memory (EM) and terminal effector (TE). Regarding the ϒ/δ T cells, although three main subtypes have been described (Vδ1, Vδ2, and Vδ3) (Kalyan and Kabelitz, 2013), we only selected two groups of ϒ/δ T cells according to their expression of Vδ2. This choice was driven by the fact that Vδ1 and Vδ3 are highly heterogeneous and their exact features are not yet well defined. Some cells positive for Vδ2 show negative expression for the TCR ϒ/δ. This might be due to steric hindrance and therefore they were still considered as Vδ2 cells. Mucosal associated invariant T (MAIT) cells were defined by the simultaneous expression of two markers, Vα7.2 and CD161.

For panel 3, we used the markers IgD and CD27 to discriminate between maturation stages of B cells: naive, non-switched memory (NSM), switched memory (SM) and exhausted memory (Ex) (Adlowitz et al., 2015). To discern plasmablasts (the memory B cells that actively produce antibodies) we used the marker CD38 (Fink, 2012). To retrieve progenitor cells, we gated on the high expression of CD34 and low expression of CD45.

For panel 4, we used different expression level of CD14 and CD16 to distinguish classical, intermediate and non-classical monocytes (Ziegler-Heitbrock et al., 2010). The DCs were classified in two main subtypes which originate from different progenitors: myeloid DCs (mDCs) and plasmacytoid dendritic cells (pDCs). These cells are antigen presenting cells and both express HLA-DR. Further gatings were performed on CD11c for mDCs and on CD123 for pDCs. NK cells express different combinations of CD16 and CD56 according to their maturation stages. However, given the low percentage of some NK subtypes, we decided not to subdivide NK cells into finer subtypes. Because CD11c is not only expressed on mDCs but also on monocytes, CD11c can also be used to better separate NK cells from non-classical monocytes. Lastly, LD granulocytes can be gated using markers already used for other cell types. LD neutrophils have been selected as producing large scattered light and high expression of CD16, and basophils have been selected from their expression of CD123 and lack of expression of HLA-DR.

For the vaccine cohort samples, one staining panel was designed to immunophenotype the major immune cell types with a focus on B lymphocytes (panel 5; Table S1).

#### Blood processing

BD Vacutainer® mononuclear Cell Preparation Tubes (CPT™; Becton Dickinson) were used for blood collection (8 ml/CPT™). The tubes were then centrifuged for 20 min at 1650 relative centrifugal force (RCF) with no brake, the plasma was removed and the PBMC layers were transferred to falcon tubes, as per the manufacturer’s instructions. The cells were washed with phosphate-buffered saline (PBS)/5% fetal bovine serum (FBS) buffer solution for 5 min at 340 R*CF.* After re-suspension, the cells were counted using a haemocytometer and split according to the downstream experiment. At this stage, aliquots of ∼5x10^6^ PBMCs were taken from the samples of the S13 and vaccine cohorts, and lysed in 1mL TRIzol® or 1 mL mirVana (Thermo Fisher Scientific), respectively. The aliquots were stored at −80°C.

#### Antibody staining and immunophenotyping

After PBMC isolation, aliquots of 1x10^6^ cells were stained with each antibody panel. The antibody clones were purchased from BioLegend, BD Biosciences or Miltenyi Biotec (Table S1). For CCR7 staining, we used clone G043H7 with a pre-incubation step at 37°C for 10 min; this clone provided a better staining index compared to the previously suggested clone 150503 (Maecker et al., 2012). All other antibodies were incubated at 4°C for 25 min. After incubation with fluorescence-conjugated antibodies, the cells were washed and re-suspended in a PBS/5% FBS/2 mM EDTA buffer solution. Single stained and unstained beads were used to establish the compensation matrix. Immunophenotyping was performed using a BD Symphony® for the S13 cohort (panel 1-4; Table S1) and a BD Fortessa® for the vaccine cohort (panel 5; Table S1). Flow cytometry data were compensated using *FACSDiva* software, quality checked with the R package *flowAI* (Monaco et al., 2016) and gated using *FlowJo* software. One flow cytometry file of the S13 cohort did not pass the quality check and hence the immunophenotyping information for the corresponding donor was excluded from further analyses.

#### FACS Sorting

From the S4 cohort, ∼2-3x10^8^ PBMCs were separated into CD3+ and CD3- populations using magnetic beads (Figure 1). The CD3+ fraction was then split into two equally sized aliquots for T cell staining with either antibody panel 1 or 2. The CD3- fraction was also split into two aliquots: one aliquot (60%) for B cell and progenitor-cell staining with panel 3, and one aliquot (40%) for monocyte, DCs, NK cells and LD granulocyte staining with panel 4. After staining, the immune cells were sorted using a BD Influx for panel 1 and 3, a FACS Aria 5 for panel 2, and a FACS Aria 4 for panel 4 (all BD Biosciences). All cells were stained and sorted within 7 h after blood collection and kept on ice between processing steps. Sorting was performed to > 98% purity and then cells were lysed in TRIzol® reagent (Thermo Fisher Scientific) and stored at −80°C.

#### RNA extraction and quantification

Total RNA was extracted from all samples (immune cell types from the S4 cohort and PBMCs from the S13 and vaccine cohorts) for gene expression analysis. The RNA from the samples of the S4 and S13 cohorts was extracted with the TRIzol® isolation protocol followed by QIAGEN RNeasy Micro clean-up procedure. The RNA from the vaccine cohort samples was isolated using a mirVana™ miRNA Isolation Kit (Thermo Fisher Scientific). RNA quality was assessed on an Agilent 2100 Bioanalyzer and samples with an RNA Integrity Number (RIN) < 6 were excluded from further analyses. The RIN for two samples of CD4 T terminal effector (T_TE_) cells was not available as the total RNA obtained was too low; these cells were excluded from further analyses. The RNA concentration was determined using a Quant-iT™ RiboGreen® RNA Assay Kit (Thermo Fisher Scientific).

#### RNA-Seq and microarray data generation

RNA aliquots from immune cell types isolated from the S4 cohort and from the PBMCs isolated from the S13 cohort were used for RNA-Seq analysis on an Illumina HiSeq 2000. The cDNA libraries were prepared from 2 ng total RNA and 1 μL of a 1:50,000 dilution of external RNA control consortium (ERCC) spike-in control mix (Thermo Fisher Scientific) using the SMARTSeq v2 protocol (Picelli et al., 2014) with the following modifications: 1) use of 20 μM template-switching oligos (TSO), 2) use of 250 pg of cDNA with 1:5 reaction mixtures of the Illumina Nextera XT kit. The length distribution of the cDNA libraries was monitored using a DNA High Sensitivity Reagent Kit (Perkin Elmer). All samples were subjected to an indexed paired-end sequencing run of 2x51 cycles (16 samples/lane). In total, 114 samples (two samples of CD4 T_TE_ cells and four samples for each of the remaining 28 immune cell types) of the S4 cohort and all 13 samples of the S13 cohort were taken forward for further analyses.

RNA aliquots from the PBMC samples obtained from the S13 and the vaccine cohorts were used for microarray analysis on an Illumina HT12 v4 BeadChip. To amplify the cDNA, the TargetAmp 2-Round aRNA Amplification Kit 2.0 (Epicenter) was used for the S13 cohort and the Illumina® TotalPrep RNA Amplification Kit (Thermo Fisher Scientific) for the vaccine cohort. The data was exported with GenomeStudio and all 13 samples of the S13 cohort and 636 samples (159 subjects with all four time points) of the vaccine cohort passed all quality checks.

### Quantification and Statistical Analysis

#### RNA-Seq and microarray data preprocessing

The genome assembly and annotation for the RNA-Seq data analysis was downloaded from GENCODE (version 26) (Harrow et al., 2012). The quality of the RNA-Seq data was assessed with *FastQC* (Andrews, 2010). The reads were pseudo-aligned to the transcriptome with *kallisto* (Bray et al., 2016), and the transcript expression values were then summarized into gene expression values with *tximport* (Soneson et al., 2016). *MultiQC* was used to assess the performance of the preprocessing steps (Ewels et al., 2016). The effect of guanine-cytosine (GC) content was explored using *EDAseq* (Risso et al., 2011). The counts were normalized for sequencing depth and gene length using the Transcripts Per Million (TPM) method (Li et al., 2010).

Microarray data were quantile normalized and corrected for batch effects using *ComBat* (Johnson et al., 2007). Illumina probes were converted into gene symbols and in the case of duplicates, only the maximum value was kept. For the cross-platform normalization of the PBMC RNA-Seq and microarray data of the S13 cohort, we selected genes with a Pearson’s correlation > 0.7 (755 genes). The UQ of the microarray values was then divided by the UQ of the RNA-Seq expression values and the resulting scaling factor was used to normalize the full microarray dataset.

#### Transcriptomics analyses

The majority of analyses on the transcriptomic data from the 29 immune cell types utilized log_2_ TPM + 1 values that were filtered only from genes with a raw count ≥ 4 in at least three samples (unless otherwise indicated). All analyses were performed within the R environment and most plots were produced with the ggplot2 package (Wickham, 2009). The *Rtsne* package and the *prcomp* function from the *stats* package were used to perform the t-SNE and PCA analyses (Figures 2A and S2A), respectively. The hierarchical clustering was generated using the *hclust* function with Euclidean distances (Figure S2B).

The transcriptomic hematopoietic tree was generated using the Spearman’s correlation coefficient (1-ρ) as pairwise distances and the neighbor-joining method for sample clustering (Figure 2B). Bootstrap values were calculated for each node to show the consistency of the branching patterns. These values were calculated by building 100 trees from randomly sampled genes with replacement and retrieving the number of times each branch conserved the topology of the consensus tree. The tree and bootstrap values were generated with the *ape* package (Paradis et al., 2004).

The DEGs were found using the *limma* package (Ritchie et al., 2015) from both TPM and TPM_TMM_ values (Table S2). The mean-variance relationship was modeled with the *voom* function and the Benjamini-Hochberg method (Benjamini and Hochberg, 1995) was used to adjust for multiple hypothesis testing. For the design matrix, each cell type was contrasted against the remaining samples. The PBMC samples were only included for linear model fitting, but they were excluded from any contrast. The differential expression analysis just described were not only applied to the 29 immune cell type classification, but also to broader categories (Table S2).

The modules of DEGs were retrieved from the differential expression analysis on TPM values (Figure 3 and Table S3). The results from all contrasts were included and a stringent threshold was used for the initial filtering (log_2_ fold change > 4 and FDR < 0.005). Modules were found using hierarchical clustering with Euclidean distance and the function *cutreeDynamic* from the R package *dynamicTreeCut* (Langfelder et al., 2008). To find the co-expressed modules (Figures S3 and S4 and Table S3), we filtered out the genes with a total log_2_ TPM + 1 expression < 50 from the 114 samples of the 29 immune cell types and kept the genes with an expression > 3.5 in at least 5 samples. Unsigned Spearman correlation was calculated for each pair of genes and the adjacency matrix was retrieved by exponentiating everything to the power of 6. The function *TOMsimilarity* from the *WGCNA* package was then used to calculate the topological overlap matrix. The hierarchical clustering was performed on the dissimilarity matrix and the *cutreeDynamic* function was then used to retrieve the modules (Langfelder and Horvath, 2008).

The heatmaps were produced with the *ComplexHeatmap* package (Gu et al., 2016). We used the gene ontology (GO) database for the enrichment analysis of the DEGs and co-expression modules (Table S3) and the Reactome databases V61 (Fabregat et al., 2016) for the DEGs of each cell type obtained using TPM_TMM_ values. (Table S3). We performed a hypergeometric test for the enrichment analysis using the overlapping genes between our gene annotation and the database (i.e., GO or Reactome) as background.

We used two approaches to compare the gene expression profiles of the 29 immune cell types of the S4 cohort. The first approach (Figure 4) consists of grouping the samples according to the FACS panel they belong to. Then we averaged the log_2_ TPM + 1 value of the samples of the same cell type and we kept the top 1000 variable genes for each group. From external datasets (Abbas et al., 2005, Calderon et al., 2018, Javierre et al., 2016, Novershtern et al., 2011), we retrieved the readily available normalized expression values and averaged the ones from samples of the same cell type. Our expression values were then compared with the expression values of selected cell types from the external datasets with the Spearman correlation. The second approach (Figure S9) consists of calculating the overlap between the genes found to be specific for our 29 immune cell types from the DEG analysis (log_2_ fold change > 2 and FDR < 0.05) and the genes specific for the cell types of the external datasets as reported in the supplementary material of the respective papers (Abbas et al., 2005, Becht et al., 2016, Bindea et al., 2013, Villani et al., 2017).

#### Normalization for mRNA abundance

We used four methods to calculate scaling factors to normalize for mRNA composition: 1) dividing the RNA quantification values obtained with the Quant-iT assay by the FACS enumeration (RNA_FACS_), 2) using our method based on deconvolution and optimization (RNA_RLM_) (see Deconvolution section), 2) inverting the trimmed mean of M-values calculated from TPM values (RNA_TMM_) (see rationale below), 3) inverting the median values of HK genes calculated from TPM values (RNA_HK_).

TPM values of the S4 cohort were normalized for mRNA abundance by multiplying them with the scaling factors just described, hence obtaining TPM_FACS_, TPM_RLM_, TPM_TMM_, TPM_HK_. When a tilde is added on top of the method subscript, e.g.${\text{ TPM}}_{\tilde{\text{RLM}}}$, it indicates that the median scaling factor was used for all samples of a specific cell type. Without tilde, the scaling factor is specific for each sample. To make the methods comparable we used PBMC samples as the reference, i.e., the mRNA scaling factors for PBMCs were always 1.

#### Rationale for the TPM_TMM_ normalization method

The trimmed mean of M-value (TMM) is an RNA-Seq normalization method implemented in the edgeR package developed to account for RNA composition. It was reported by Robinson and Oshlack (2010) and it has been thoroughly described by Maza (2016).

Robinson and Oshlack state that normalizing for library size is a sufficient practice for technical replicates (step I of Table 2 in Maza 2016). The resulting values could then be multiplied by 1 million to obtain the count per million (CPM):

$$\frac{Raw\;counts}{library\;size}\;x\;10^{6}$$

However, this approach is not appropriate for several situations where the biological samples have different RNA composition. In a similar way to previous normalization approaches developed for microarray data, the assumption behind TMM is that the majority of genes are not differentially expressed and hence they should have the same distribution. This is an accepted practice for the most common analysis done on gene expression data, i.e., finding of DEGs. The library size multiplied by the TMM values give the effective library size (step V in Maza 2016):

effectivelibrarysize=librarysizexTMM

Hence, the effective library size should be used to normalize the raw counts to account for RNA composition (step VII in Maza 2016):

$$\frac{Raw\;counts}{effective\;library\;size}\;x\;10^{6}$$

The alternative approach described in our paper to normalize for RNA composition is to use TPM values, which are normalized by transcript gene length and whose library size is always 10^6^, scaled by an mRNA abundance scaling factor. Hence:

TPMxscaledmRNAabundance

If we use TPM values as raw counts for the calculation of the TMM values, we have:

$$\frac{TPM}{10^{6}\;x\;TMM}\;x\;10^{6}$$

Assuming that TMM values should normalize for RNA composition we can then state that both normalization approaches are equivalent:

$$TPM\;x\;scaled\;mRNA\;abundance\;\approx \;\frac{TPM}{10^{6}\;x\;TMM}\;x\;10^{6}$$

that is reduced to:

$$scaled\;mRNA\;abundance\;\approx \;\frac{1}{TMM}\;$$

#### Retrieval of mRNA scaling factor through RLM deconvolution and optimization

To retrieve the mRNA scaling factors, we used RLM since the method is more resilient to noise. However, the concept can be more easily described with an LM framework as:

$$y={\hat{\text{β}}}_{1}x_{1}+{\hat{\text{β}}}_{2}x_{2}+\ldots +{\hat{\text{β}}}_{\text{n}}x_{n}+\varepsilon$$

where **y** is the expression of one gene in a set of heterogeneous samples (in our case PBMCs), **x**_**1**_,**x**_**2**_,…,**x**_**n**_, are the gene expression values of the same gene in each constituting cell type, and β_1_, β_2_,…, β_n_, are the coefficients describing the change in **y** with respect to **x**. Bold characters indicate vectors of numbers, while regular characters indicate scalars. In this model, there is no intercept term because the regression model is forced to pass through the origin. In other words, when all the predictor variables (the expression in the immune cell types) are 0, the response variable (the expression in the PBMCs) must be 0.

When the gene expression values are correctly normalized and hence correspond to the real absolute gene expression, the β coefficient corresponds to the immune cell proportion only. However, when the gene expression values are not normalized by mRNA abundance (i.e. TPM values), the β coefficients account for both immune-cell proportion and mRNA abundance. In this case, the model can be re-written as:

$$\begin{array}{cc} y={\hat{\text{ρ}}}_{1}{\hat{\text{α}}}_{1}x_{1}+{\hat{\text{ρ}}}_{2}{\hat{\text{α}}}_{2}x_{2}+\ldots +{\hat{\text{ρ}}}_{\text{n}}{\hat{\text{α}}}_{\text{n}}x_{n}+\varepsilon , & \{\begin{array}{c} \hat{\text{ρ}}\;>0\\ \hat{\text{α}}>0 \end{array} \end{array}$$

where the $\hat{\text{ρ}}$ values account for the proportions, the $\hat{\text{α}}$ values account for mRNA abundance of each cell type and both the $\hat{\text{ρ}}$ and $\hat{\text{α}}$ values are positive numbers. We cannot estimate both the $\hat{\text{ρ}}$ and $\hat{\text{α}}$ values with the gene expression values only; however, we can estimate the $\hat{\text{α}}$ values by knowing real cell-type proportions (ρ), that in our case have been calculated by flow cytometry. To obtain an optimal $\hat{\text{α}}$ value for each cell type, we used an optimization algorithm to find the $\hat{\text{α}}$ value that minimizes the root mean square error (RMSE) between the estimated $\hat{\rho }$ and real **ρ** proportions over a set of k individuals (in our case the S13 cohort). Hence, for each cell type:

$$\underset{\hat{\text{α}}\in \left(\text{l},\text{u}\right)}{\min}\sqrt{\sum_{\text{i}=1}^{\text{k}}{\left({\hat{\text{ρ}}}_{\text{i}}-{\text{ ρ}}_{\text{i}}\right)}^{2}}$$

where l and u are the lower and upper limits for the $\hat{\text{α}}$ value, respectively, and can be optionally set using prior knowledge. For the optimization procedure, we used the *optimize* function from the *stats* package, which uses a combination of golden section search and successive parabolic interpolation.

#### Suitable cell types and signature matrices for absolute deconvolution

Deconvolution methods only work for cell types that have a detectable and specific signal pattern from a heterogeneous sample. Hence, when possible we merged cell types from the classification used for FACS (i.e., the 29 cell types) with no detectable and/or no specific signal into broader cell types. To choose the most detailed and well-performing cell type classification for deconvolution, we performed two exhaustive searches. The first exhaustive search consisted of using all the cell type combinations derived by merging the cell types from our four largest lineages: CD4 T cells, CD8 T cells, B cells and Monocytes. Then, we delineated 9 cell type classifications which include the cell types that gave the best results in the first exhaustive search (Table S2). For the second exhaustive search, we used all possible combinations from the 9 cell classifications. From the results obtained we then picked the most effective cell type classification (Figures 6A and S8A and Table S5). From this procedure we selected a set of 17 and 11 cell types for RNA-Seq and Microarray deconvolution, respectively.

In order to get the signatures of the immune compartments we calculated median TPM values for the 29 sorted cell types and median TPM values weighed by flow cytometry proportions for the merged cell types. The genes compiling the signature matrix were selected using the results of the differential expression analysis on the TPM values between each cell type and all the remaining samples. We ranked the genes by their q value (false discovery rate) and kept those with a log_2_ fold change > 2 and q-value < 0.05. Optionally, a set of filtering procedures was performed to remove noisy genes, i.e., those with very high expression (> 5000 in at least one cell type), very low expression (sum of all samples < 50) and poor specificity (log_2_ fold change > 0.2 between first and second highest expression). To further reduce the number of genes to include in the signature matrix, we calculated the condition number (kappa) over matrices of increasing size and selected the matrix with the lowest kappa. After retrieval of the mRNA scaling factors described in the next paragraph, the values were normalized for mRNA abundance.

To estimate cell type proportions, we used robust linear modeling (RLM) and signature matrices with low condition numbers for a set of 17 and 11 cell types (as found by the exhaustive searches described previously) for RNA-Seq and microarray data, respectively (Figures 6B and S8B). The signature matrix for microarray data was also filtered for noisy genes as described previously. The two signature matrices normalized by mRNA abundance, ABIS-Seq and ABIS-Microarray, can be directly used to deconvolute PBMC transcriptomic data (Table S5). If using RNA-Seq, the gene expression values should be TPM. If using microarray, the dataset should be reduced to only the genes present in the ABIS-Microarray signature matrix and the “target quantiles microarray” sheet from Table S5 should be used for quantile normalization.

#### Absolute deconvolution validation

Three external datasets were collected to validate our signature matrices (for RNA-Seq and microarray deconvolution) (Figure S9). 1) The dataset from Zimmermann et al. (2016) provides both flow cytometry data and RNA-Seq data and the data are available through ImmPort (http://www.immport.org) with accession number SDY67. Cell types proportions were retrieved from their B cell, T regs and innate flow cytometry panels. 2) The dataset from Newman et al. (2015) was downloaded from GEO: GSE65133. The dataset provides both microarray data and cell type proportions. 3) For the dataset of Mohanty et al. (2015), we downloaded the microarray data from GEO: GSE9654, and flow cytometry data from ImmPort, accession number SDY404. We analyzed the T cells and B cells panels (L1 and L4, respectively).

The three external datasets contain both PBMC transcriptomic data and flow cytometry data where cell type proportions can be obtained in relation to the PBMC fraction of blood. All the flow cytometry proportion extracted are available in Table S6.

### Data and Software Availability

The accession number for the RNA-Seq data of the 29 immune cell types of the S4 cohort and PBMCs of the S13 cohort is GEO: GSE107011. The microarray data of the PBMCs of the S13 cohort is available from GEO: GSE106898. Both mentioned GEO repositories are accessible from the SuperSeries GEO: GSE107019. The microarray data from the vaccine cohort is available from GEO: GSE107990.

A shiny application to perform absolute deconvolution is available from https://github.com/giannimonaco/ABIS.

## Supplemental Information

Document S1. Figures S1–S9

Table S1. Antibody Panels Used for Our S4, S13, and Vaccine Cohort and Cell Type Properties of the Sorted 29 Immune Cell Types, Related to Figure 1 and STAR Methods

Table S2. Fold Change and FDR Values of the DEGs Found Using TPM and TPM_TMM_ Values, Related to Figure 3 and STAR Methods

Table S3. Gene Modules and Functional Enrichments Analysis, Related to Figures 3 and S3–S5 and STAR Methods

Table S4. DEGs of PBMC of the Vaccine Cohort between Day 0 and Day 2, Day 7, and Day 28, Related to Figures S3 and S4 and STAR Methods The genes overlapping with the ones of the modules of the co-expression heatmap in Figure S4 are indicated.

Table S5. Well-Conditioned Signature Matrices for RNA-Seq (ABIS-Seq) and Microarray (ABIS-Microarray) Deconvolution, Related to Figures 6 and S8 and STAR Methods It also includes the target quintiles to normalize PBMC microarray samples (only the genes which constitute the ABIS-Microarray signature matrix).

Table S6. Cell Type Proportions with Respect to PBMCs Samples Collected from Our S13 Cohort, Our Vaccine Cohort, Zimmermann et al. (2016), and Mohanty et al. (2015), Related to Figures 6, S8, and S9 and STAR Methods

Document S2. Article plus Supplemental Information
