# Supplementary material for: RNA-Seq Signatures Normalized by mRNA Abundance Allow Absolute Deconvolution of Human Immune Cell Types
Source: Cell Rep. 2019 Feb 5;26(6):1627–1640.e7. doi: 10.1016/j.celrep.2019.01.041 (PMC6367568; doi:10.1016/j.celrep.2019.01.041)
Supplement: Document S1. Figures S1–S9 [file mmc1.pdf]

Cell Reports, Volume *26*

Supplemental Information

RNA-Seq Signatures Normalized by mRNA Abundance Allow Absolute Deconvolution of Human Immune Cell Types

Gianni Monaco, Bernett Lee, Weili Xu, Seri Mustafah, You Yi Hwang, Christophe Carré, Nicolas Burdin, Lucian Visan, Michele Ceccarelli, Michael Poidinger, Alfred Zippelius, João Pedro de Magalhães, and Anis Larbi

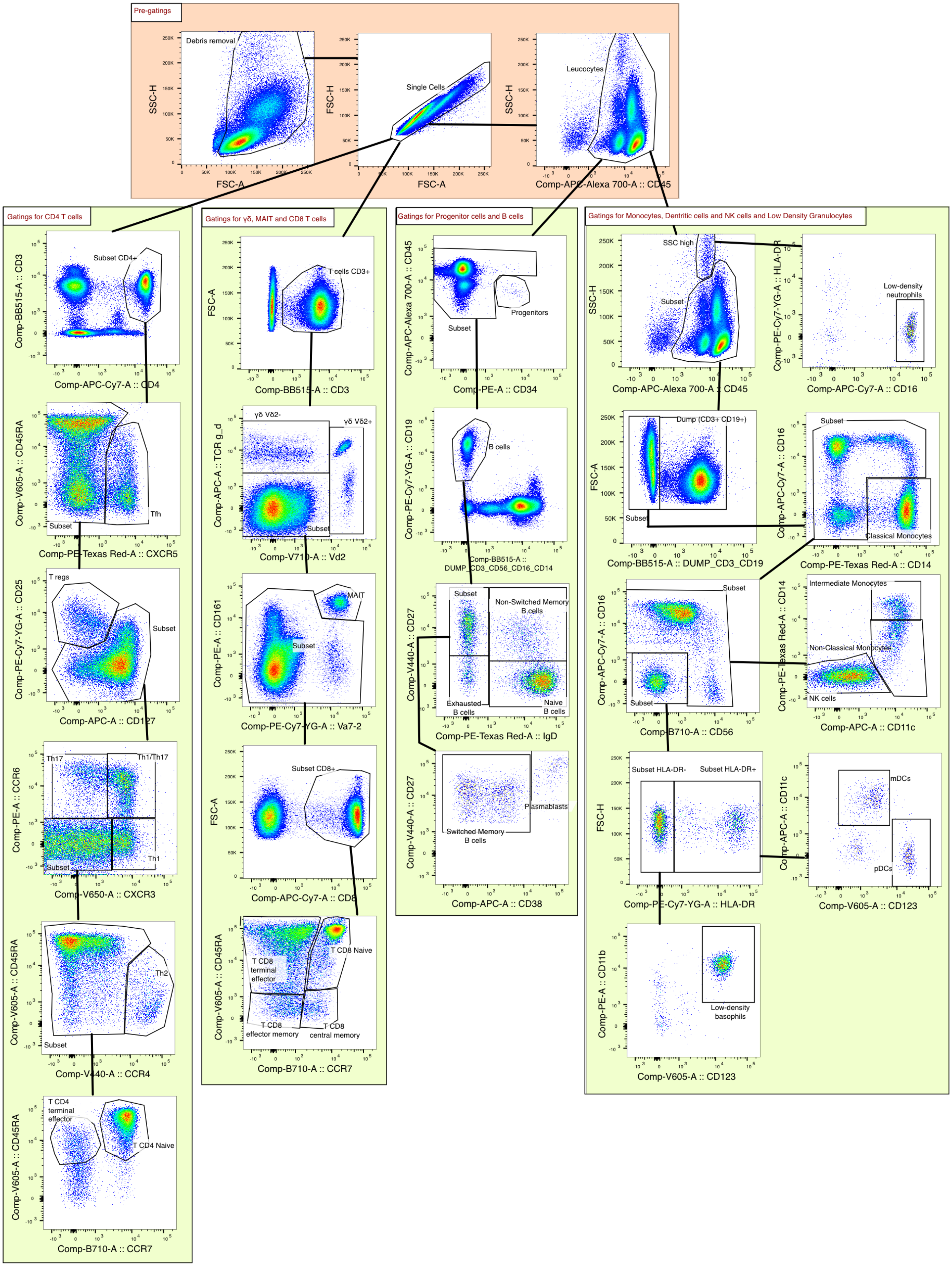

Figure S1 Gating strategies for cell sorting and immunophenotyping of the 29 immune cell types from the S4 and S13 cohort. Related to Figure 1, Table S1 and STAR Methods.

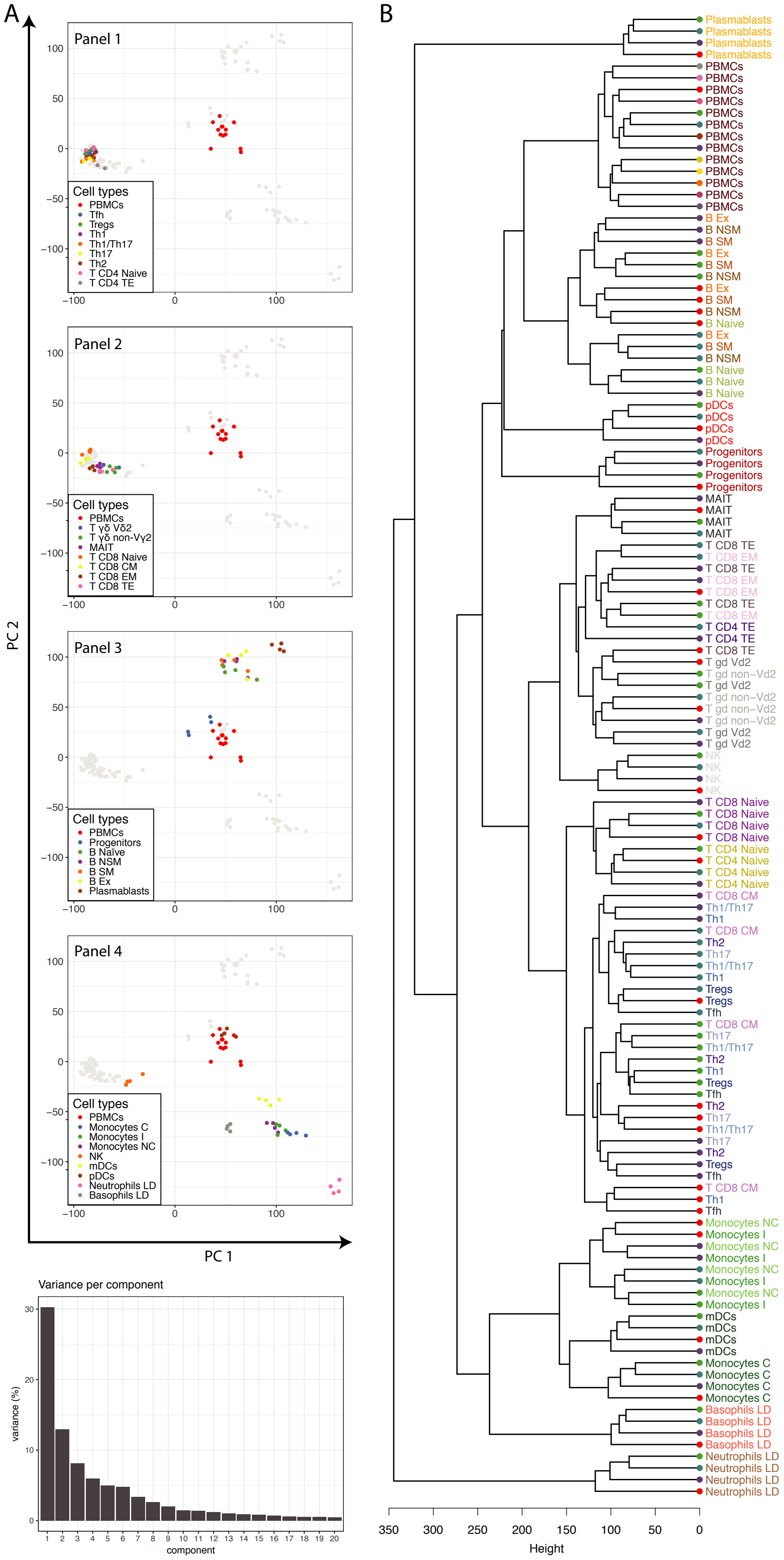

Figure S2 PCA analysis and hierarchical clustering of TPM values. Related to Figure 2 and STAR Methods.
(A) PCA analysis showing the first two components and the variance explained by the first 20 components.
(B) Hierarchical clustering of the immune cell types. The colored dots at the end of each terminal node correspond to different individuals.

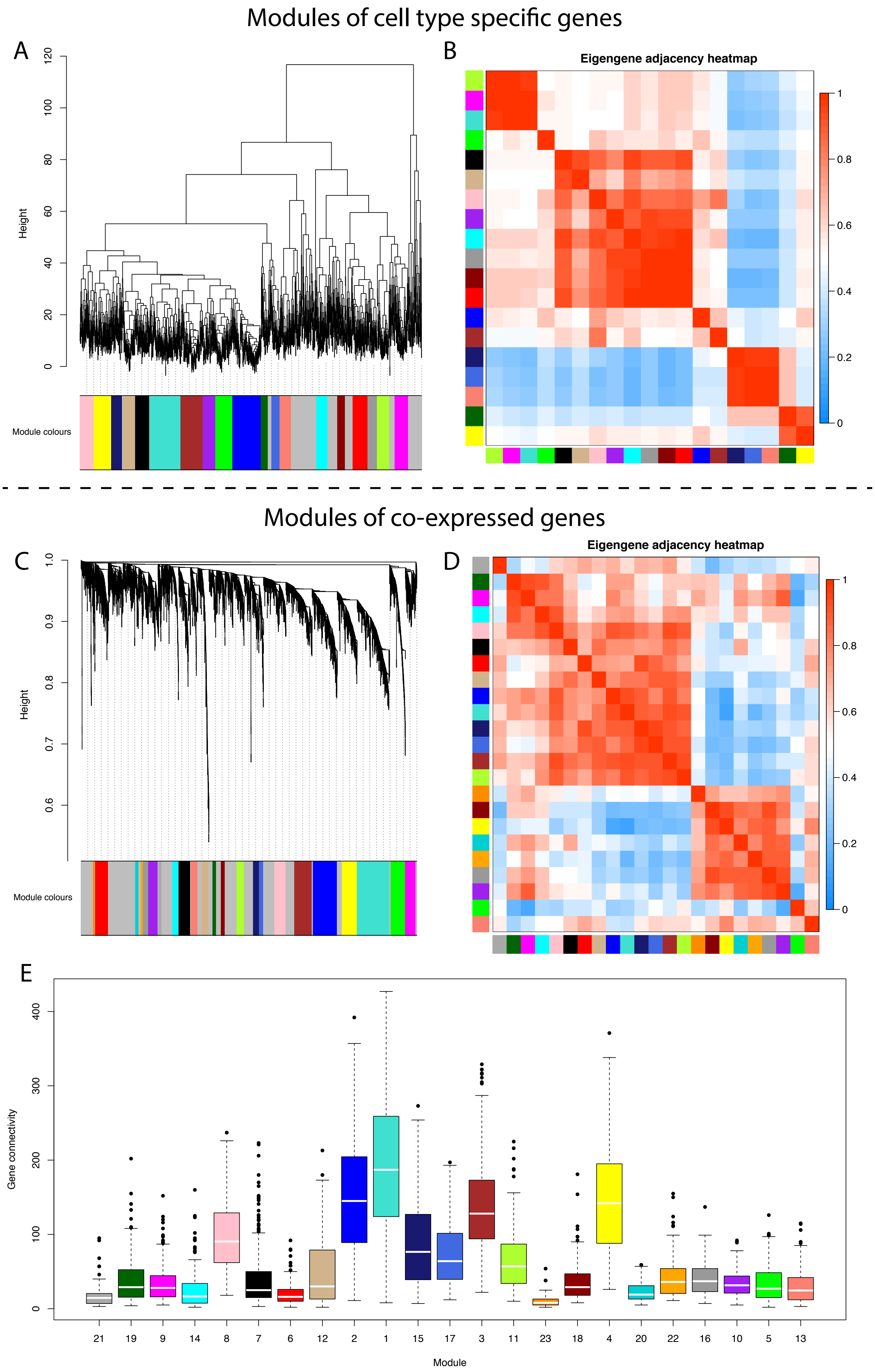

Modules of cell type specific genes
A
B
Eigengene adjacency heatmap
Height
Module colours
Modules of co-expressed genes
C
D
Eigengene adjacency heatmap
Height
Module colours
E
Gene connectivity
Module
21
19
9
14
8
7
6
12
2
1
15
17
3
11
23
18
4
20
22
16
10
5
13

Figure S3 Module analysis of the cell type specific (A-B) and co-expression (C-E) modules. Related to Figures 3 and S4, Table S3 and STAR Methods.

(A) Hierarchical clustering of the DEGs generated using Euclidean distances. The modules were retrieved by cutting the tree with the *hybrid* method from the Dynamic Tree Cut algorithm.

(B) Eigengene adjacency heatmap of the modules reported in (A).

(C) Hierarchical clustering generated using the "unsigned" adjacency matrix created in two steps as described in the *WGCNA* manual. In the first step, we calculated the absolute Spearman's correlation of each gene pair, which was then raised to the soft thresholding power of 6 to approximate the scale-free topology. In the second step, we calculated the consensus topological overlap that has been then used for clustering. The modules were retrieved by cutting the tree with the *hybrid* method from the Dynamic Tree Cut algorithm.

(D) Eigengene adjacency heatmap of the modules reported in (C).

(E) Boxplot of the co-expression connectivity of the genes contained in each module. The colors of the boxplots correspond to the colors of the modules generated with *WGCNA*.

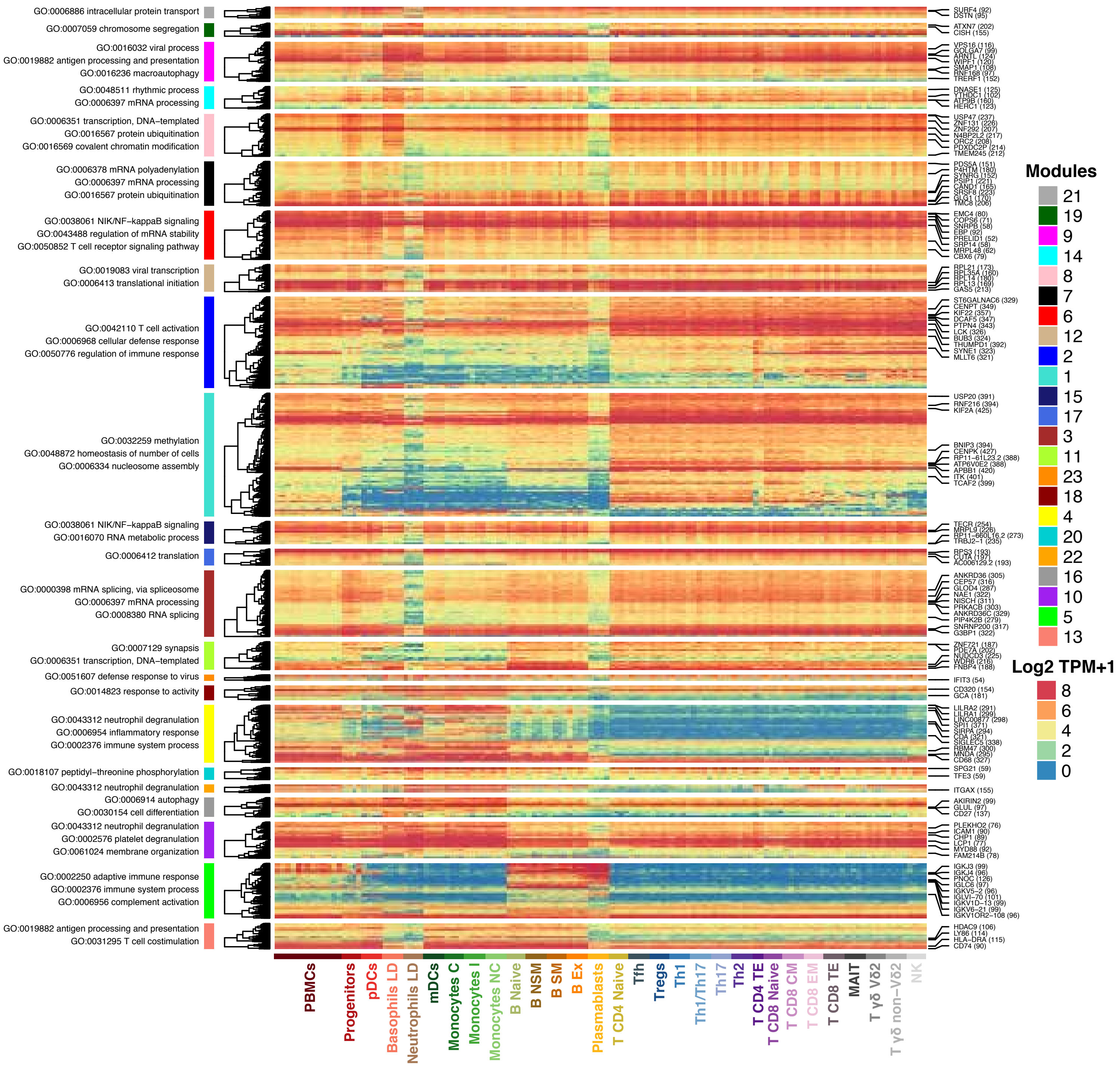

Figure S4 Heatmap of co-expression modules. Related to Figure 3 and S3 and STAR Methods.
The co-expression modules have been found using the WGCNA package on TPM values. The most relevant gene ontology terms associated with each module are reported on the left. For each module, the genes with higher intra-modular connectivity are reported on the right (Full list in **Table S3**). See **Figure S3** for details on module selection.

A

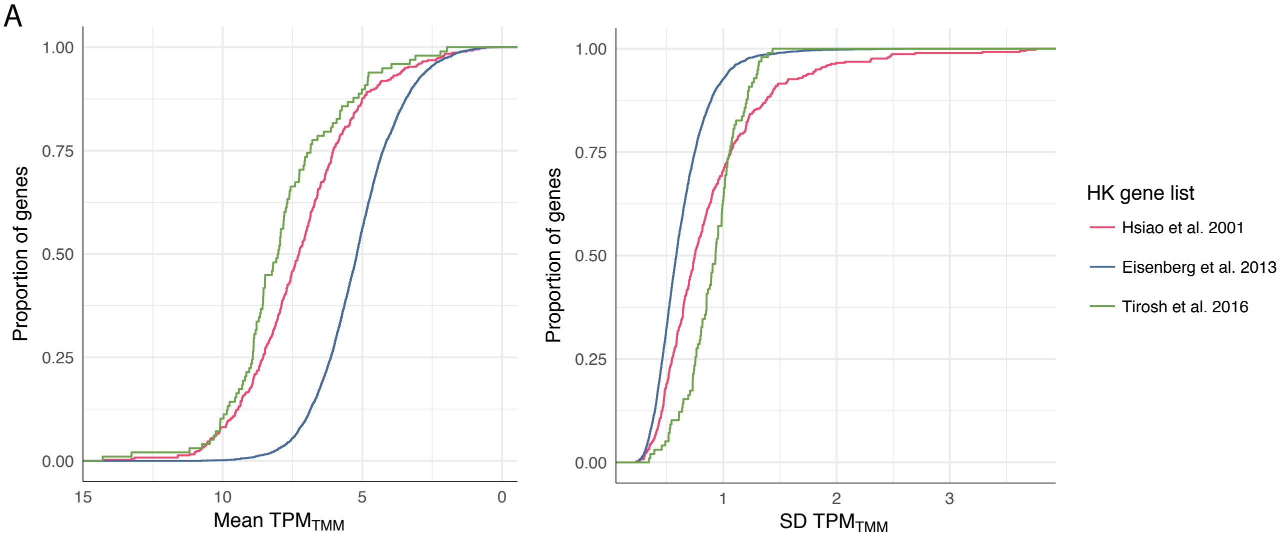

B

HK list	Size list	% overlap with our annotation	% no expression in any immune cell	% with mean > 5 and SD < 1 in our data
Hsiao et al. 2001	389	0.992	0.018	0.573
Eisenberg et al. 2013	3804	0.966	0.013	0.506
Tirosh et al. 2016	98	0.969	0.051	0.48

Figure S5 Analysis of housekeeping (HK) genes from previous works and overlap with our data. Related to Figure 3 and Table S3. (A) Empirical cumulative density functions (ECDFs) showing the proportion of genes from 3 housekeeping (HK) genes lists with TPM_{TMM} values at decreasing mean and increasing standard deviation (SD). Roughly, 75% of HK genes have a mean > 5 and a SD < 1. (B) The proportion of genes in the three HK gene lists that overlap with our annotation (third column of the table), that are not expressed in any immune cells (fourth column) and that have a TPM_{TMM} values expression with a mean > 5 and SD < 1 (fifth column). We considered a gene to be expressed when it had a raw count ≥4 in at least three samples from a total of 127 samples (composed of the 29 immune cell types and PBMCs).

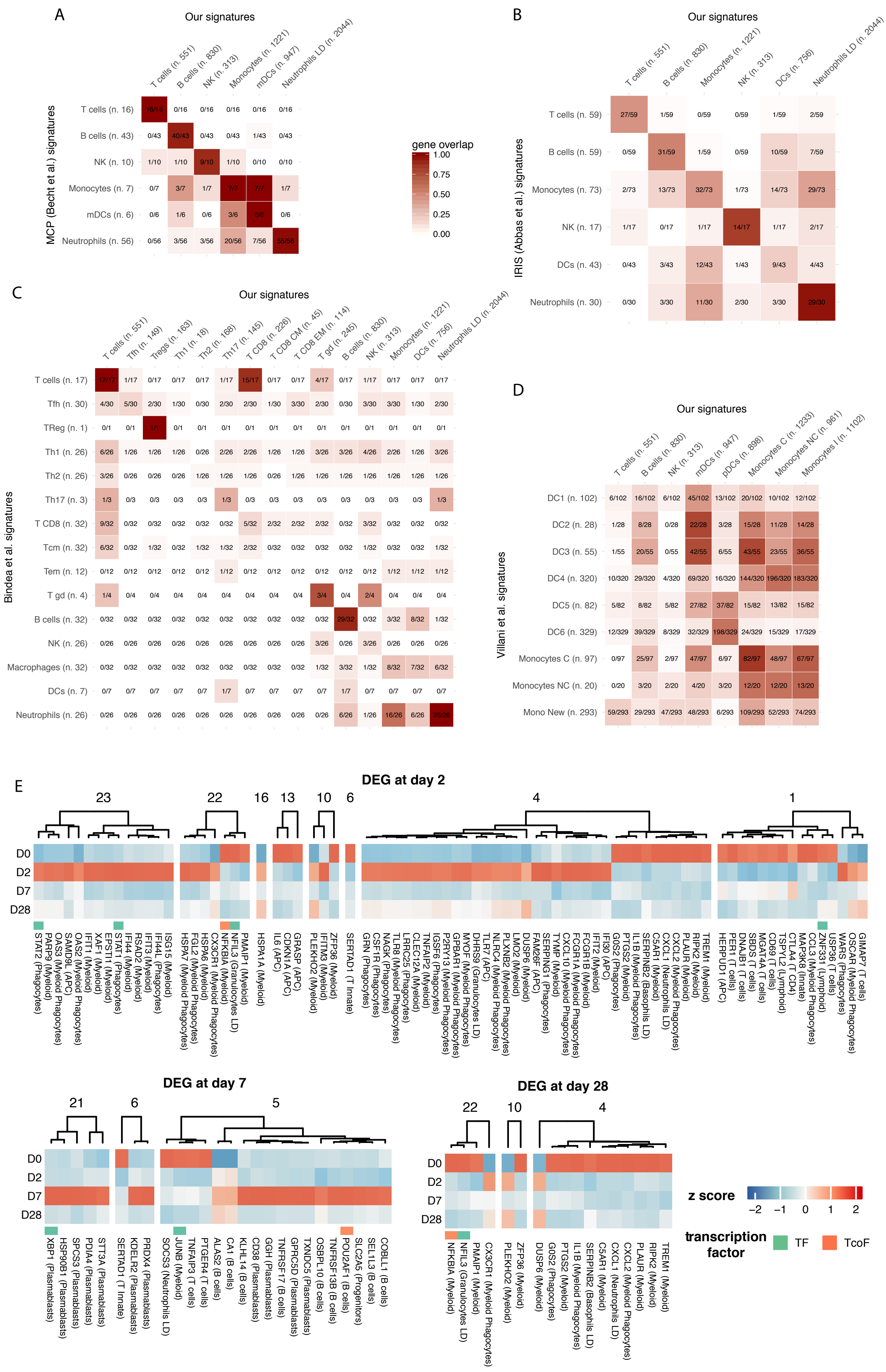

A
Our signatures
MCP (Becht et al.) signatures
gene overlap
B
IRIS (Abbas et al.) signatures
C
Bindea et al. signatures
D
Villani et al. signatures
E
DEG at day 2
DEG at day 7
DEG at day 28
z score
transcription factor
TF
TcoF

Figure S6 Validation of our gene expression profiles and gene module analysis. Related to Figure 4, Table S4 and STAR Methods.
(A-D) Validation of our cell types specific genes with ones from external datasets. Our signatures (columns) are the differentially expressed genes with log fold change > 2 and FDR < 0.05 between one cell type and all remaining immune samples. The signatures of external datasets have been taken from supplementary files of the respective papers. The heatmaps show the number of genes of the signatures of external datasets that overlap with our signatures. Only relevant cell types have been chosen for the visualization. The comparison was done with Becht *et al.* (2016) in (A), with Abbas *et al.* (2005) in (B), with Bindea *et al.* (2013) (C), and with Villani *et al.*, (2017) in (D). In (D), the DC2 and DC3 should correspond to mDCs and the DC6 to pDCs.
(E) Analysis of a microarray dataset on PBMC samples (vaccine cohort) using our RNA-Seq transcriptomic resources. Samples of the vaccine cohort were collected at four time points following flu vaccination (0, 2, 7 and 28 days abbreviated as D0, D2, D7 and D28, respectively). Three heatmaps show differentially expressed genes at days 2, 7 and 28 versus day 0 that enrich co-expression modules (numbers above the dendrograms) reported in **Figure S4** and **Table S3**. The specificity of each gene retrieved though our differential expression analysis (**Table S2**) is reported in parenthesis. Genes listed as transcription factors and co-factors in the AnimalTFDB database are annotated.

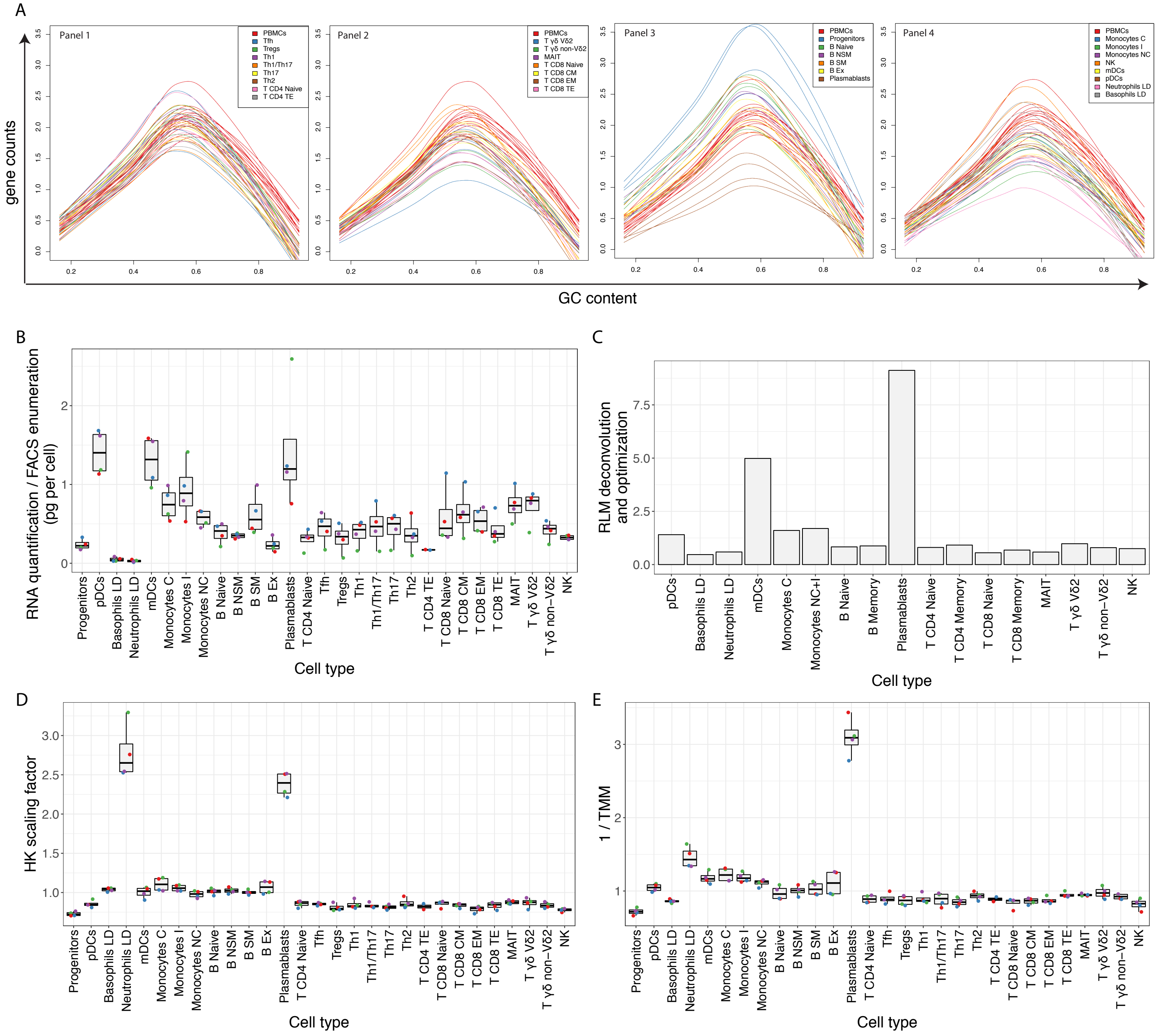

Figure S7 Transcriptome composition. Related to Figure 5 and STAR Methods.

(A) Raw gene counts plotted against GC content for the PBMCs and the 29 immune cell type samples of the S4 and S13 cohorts. PBMCs are reported in each plot and the color-code for all samples is equivalent to that used in **Figure 2A** and **S2A**. The effect of GC content on raw counts with the *EDASeq* tool, we found that the expression of all genes tended to increase at medium GC values, which is in agreement with the findings of the *EDASeq* developers (Risso *et al.*, 2011). Since the same amount of RNA starting material was used for the RNA-Seq profiling of the 29 cell types, a masking effect on the expression of low frequency transcripts by a few dominant highly expressed genes is noticeable in raw counts. For example, in comparison to other cell types, plasmablasts and LD neutrophils showed an overall lower GC content whereas progenitors displayed a higher GC content. We consider that these GC content effects are likely due to differences in mRNA heterogeneity.

(B-E) Scaling factors that are supposed to adjust for RNA abundance generated with different methods (in B, D and E donors are color-coded): (B) RNA yield in picograms per cell estimated by dividing the RNA quantification by the FACS enumeration; (C) mRNA abundance scaling factors obtained with the RLM deconvolution and optimization procedure (**STAR Methods**); (D) the median gene expression of housekeeping gene inverted and scaled so that the value for PBMCs is 1; (E) inverted TMM value (**STAR Methods**).

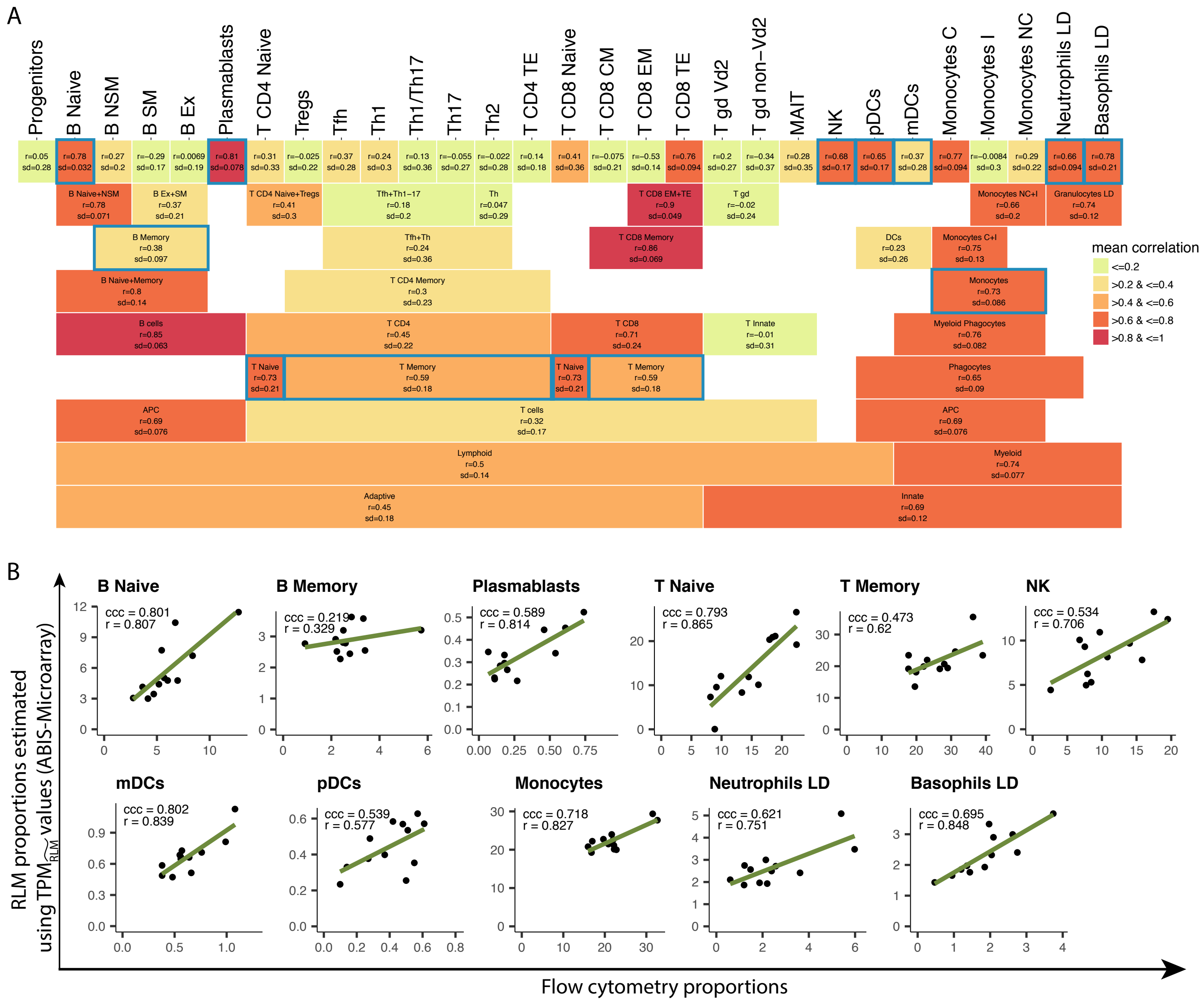

Figure S8 Absolute deconvolution of microarray PBMC samples. Related to Figure 6, Table S5 and STAR Methods.
(A) Exhaustive search performed to find the most inclusive and performant combination of cell types to use for microarray deconvolution.
(B) Comparison between real flow cytometry proportions and RLM deconvoluted proportions using microarray data for the heterogeneous PBMC sample and filtered and cross-normalized RNA-Seq data for the signature matrix (ABIS-Microarray). The concordance correlation coefficient (ccc) and the Pearson's correlation coefficient (r) are shown on each plot.

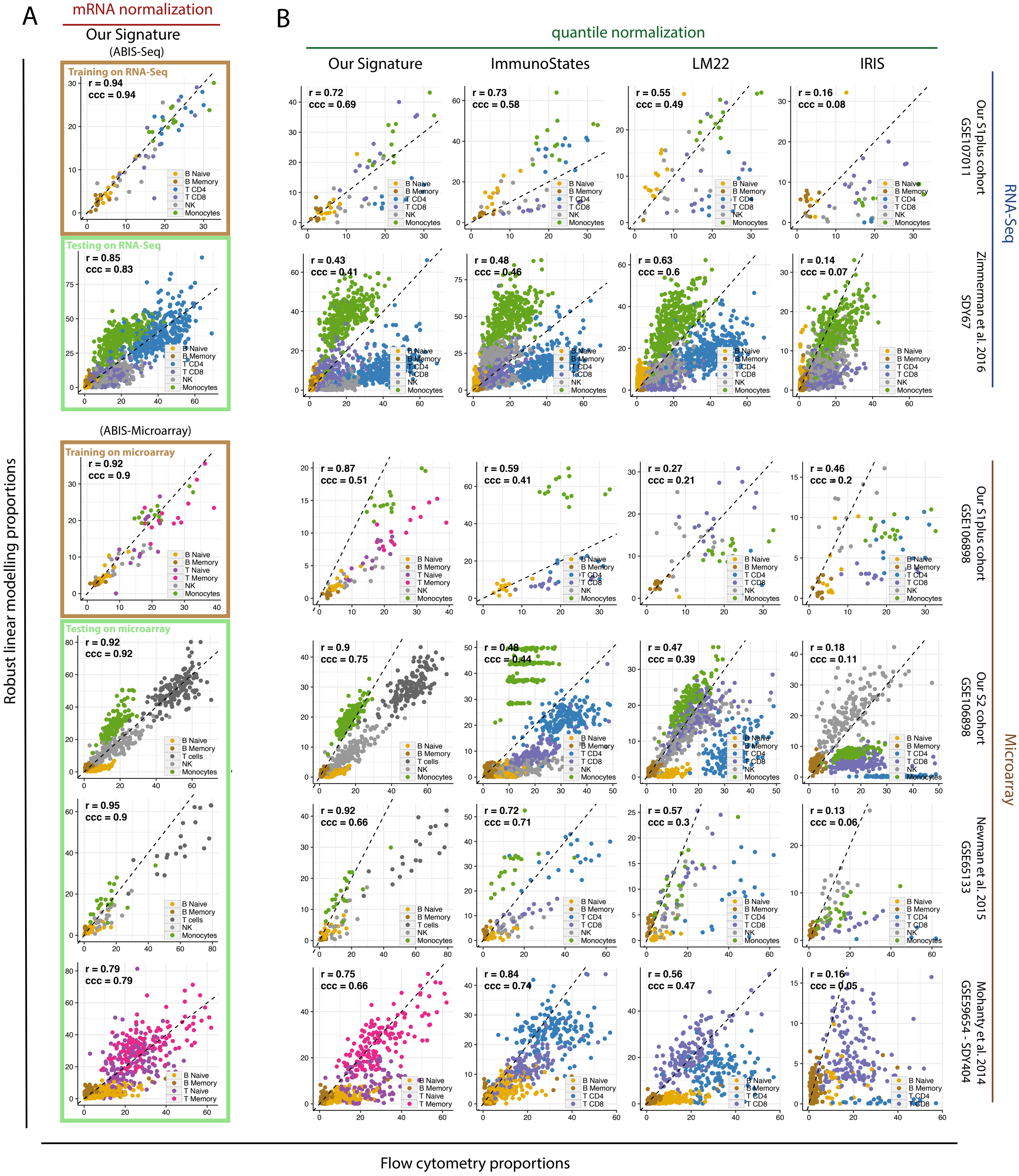

Figure S9 Validation of our signature matrices and benchmark with external ones. Related to Figure 7, Table S6 and STAR Methods.
(A) Validation of absolute deconvolution using our signature matrices normalized for mRNA abundance (ABIS-Seq and ABIS-Microarray) in one RNA-Seq dataset and three microarray datasets (green boxes in the first column).
(B) The results were compared to the ones obtained by using quantile normalization using our signature matrices and three external ones (ImmunoStates, LM22 and IRIS). Because all the datasets and signature matrices provide a different composition of cell types, we only validated the major cell types. The results shown here show a global Pearson and concordance correlation coefficient for all cell types shows in each plot.
